# Lysosomal cholesterol overload in macrophages promotes liver fibrosis in a mouse model of NASH

**DOI:** 10.1084/jem.20220681

**Published:** 2023-09-19

**Authors:** Michiko Itoh, Atsushi Tamura, Sayaka Kanai, Miyako Tanaka, Yohei Kanamori, Ibuki Shirakawa, Ayaka Ito, Yasuyoshi Oka, Isao Hidaka, Taro Takami, Yasushi Honda, Mitsuyo Maeda, Yasuyuki Saito, Yoji Murata, Takashi Matozaki, Atsushi Nakajima, Yosky Kataoka, Tomoo Ogi, Yoshihiro Ogawa, Takayoshi Suganami

**Affiliations:** 1 Department of Molecular Medicine and Metabolism, Research Institute of Environmental Medicine, Nagoya University, Nagoya, Japan; 2 Department of Bioelectronics, Institute of Biomaterials and Bioengineering, Tokyo Medical and Dental University, Tokyo, Japan; 3 Kanagawa Institute of Industrial Science and Technology, Kawasaki, Japan; 4 Department of Metabolic Syndrome and Nutritional Science, Research Institute of Environmental Medicine, Nagoya University, Nagoya, Japan; 5 Department of Organic Biomaterials, Institute of Biomaterials and Bioengineering, Tokyo Medical and Dental University, Tokyo, Japan; 6 Department of Immunometabolism, Nagoya University Graduate School of Medicine, Nagoya, Japan; 7 Institute of Nano-Life-Systems, Institutes of Innovation for Future Society, Nagoya University, Nagoya, Japan; 8 Department of Genetics, Research Institute of Environmental Medicine, Nagoya University, Nagoya, Japan; 9 Department of Gastroenterology, Yamaguchi University Graduate School of Medicine, Yamaguchi, Japan; 10 Department of Gastroenterology and Hepatology, Yokohama City University Graduate School of Medicine, Yokohama, Japan; 11 Multi-Modal Microstructure Analysis Unit, RIKEN-JEOL Collaboration Center, Kobe, Japan; 12 Laboratory for Cellular Function Imaging, RIKEN Center for Biosystems Dynamics Research, Kobe, Japan; 13 Division of Molecular and Cellular Signaling, Department of Biochemistry and Molecular Biology, Kobe University Graduate School of Medicine, Kobe, Japan; 14 Division of Biosignal Regulation, Department of Biochemistry and Molecular Biology, Kobe University Graduate School of Medicine, Kobe, Japan; 15 Department of Medicine and Bioregulatory Science, Graduate School of Medical Sciences, Kyushu University, Fukuoka, Japan; 16 Center for One Medicine Innovative Translational Research, Gifu University Institute for Advanced Study, Gifu, Japan

## Abstract

Accumulation of lipotoxic lipids, such as free cholesterol, induces hepatocyte death and subsequent inflammation and fibrosis in the pathogenesis of nonalcoholic steatohepatitis (NASH). However, the underlying mechanisms remain unclear. We have previously reported that hepatocyte death locally induces phenotypic changes in the macrophages surrounding the corpse and remnant lipids, thereby promoting liver fibrosis in a murine model of NASH. Here, we demonstrated that lysosomal cholesterol overload triggers lysosomal dysfunction and profibrotic activation of macrophages during the development of NASH. β-cyclodextrin polyrotaxane (βCD-PRX), a unique supramolecule, is designed to elicit free cholesterol from lysosomes. Treatment with βCD-PRX ameliorated cholesterol accumulation and profibrotic activation of macrophages surrounding dead hepatocytes with cholesterol crystals, thereby suppressing liver fibrosis in a NASH model, without affecting the hepatic cholesterol levels. In vitro experiments revealed that cholesterol-induced lysosomal stress triggered profibrotic activation in macrophages predisposed to the steatotic microenvironment. This study provides evidence that dysregulated cholesterol metabolism in macrophages would be a novel mechanism of NASH.

## Teaser

Lysosomal cholesterol overload triggers phenotypic changes and profibrotic activation of macrophages around dead hepatocytes containing cholesterol crystals in the development of NASH. Cholesterol excretion from macrophages using β-cyclodextrin polyrotaxane, which is designed to release β-cyclodextrin in lysosomes, alleviates liver fibrosis.

## Introduction

Nonalcoholic fatty liver disease (NAFLD) is a clinical spectrum that encompasses simple steatosis to nonalcoholic steatohepatitis (NASH), the latter of which is considered among the top etiologies for hepatocellular carcinoma and indications for liver transplantation (Younossi et al., 2018, 2019). Although simple steatosis is generally benign and reversible, 10–30% of NAFLD patients develop NASH and cirrhosis. According to the two-hit or multiple-hit hypothesis, the pathogenesis of NASH involves dysregulated lipid metabolism and inflammatory and profibrotic cues (Tilg and Moschen, 2010). Numerous clinical trials targeting each process have been conducted worldwide; however, there are no approved therapeutic strategies for NASH (Vuppalanchi et al., 2021).

The concept of “lipotoxicity” has been pointed out that cytotoxic lipids, such as cholesterol, fatty acids, and their metabolites, may directly cause cell death or act in a proinflammatory and profibrotic manner in the pathogenesis of NASH (Neuschwander-Tetri, 2010). Lipidomic analysis of human livers revealed that levels of free cholesterol are increased in NASH, while those of esterified cholesterol do not change compared to simple steatosis (Caballero et al., 2009; Van Rooyen et al., 2011). Recent evidence has shown that excessive dietary cholesterol exaggerates steatohepatitis in rodents and humans (Farrell et al., 2019; Musso et al., 2003; Savard et al., 2013). Moreover, free cholesterol is an important lipotoxic lipid inducing hepatocyte death (Gan et al., 2014; Marí et al., 2006), upregulation of profibrotic factors in hepatocytes (Wang et al., 2020), and activation of hepatic stellate cells, all of which lead to overproduction of extracellular matrix (Teratani et al., 2012; Tomita et al., 2014). Interestingly, there exist cholesterol crystals within the lipid droplets of steatotic hepatocytes in both NASH patients and NASH models (Ioannou et al., 2013, 2017). Of note, cholesterol crystals are observed in almost all the patients with fibrosing NASH but only in a few patients with simple steatosis (Ioannou et al., 2019), suggesting the pathological significance of cholesterol crystallization in the development of NASH.

We and others have reported unique histological structures termed crown-like structures (CLS), where macrophages surround and engulf dying or dead hepatocytes with large lipid droplets in murine and human NASH (Ioannou et al., 2013; Itoh et al., 2013, 2017). Activated fibroblasts and collagen deposition are observed around CLS, and the number of CLS has been positively correlated with the fibrosis area, suggesting that CLS are the site of interaction of dead hepatocytes and stromal cells, which exerts as a driving force of liver fibrosis (Itoh et al., 2013). Indeed, hepatocyte death triggers phenotypic changes in macrophages constituting CLS thereby acquiring profibrotic properties (Itoh et al., 2017). We also characterized these macrophages as a disease-specific macrophage subset with a gene expression profile distinct from other scattered macrophages (Itoh et al., 2017; Kanamori et al., 2021). However, it still remains unclear how these macrophages undergo phenotypic changes interacting with dead hepatocytes.

In this study, we focused on the lysosomal accumulation of free cholesterol and subsequent lysosomal dysfunction in CLS-constituting macrophages in our NASH model using genetically obese melanocortin 4 receptor–deficient (MC4R-KO) mice (Farrell et al., 2019; Itoh et al., 2011). We employed β-cyclodextrin polyrotaxane (βCD-PRX), a unique supramolecular compound, to excrete free cholesterol specifically from lysosomes (Tamura and Yui, 2015, 2018). When administered to MC4R-KO mice, βCD-PRX effectively ameliorated liver fibrosis at least partly by decreasing free cholesterol content in macrophages and suppressing the activation of profibrotic pathways in a NASH model, without affecting the hepatic and serum levels of cholesterol. In vitro experiments revealed that loading of cholesterol crystals induces lysosomal dysfunction and profibrotic changes in macrophages, which was reversed by βCD-PRX administration. This study demonstrates that lysosomal cholesterol overload triggers phenotypic changes and profibrotic activation of macrophages interacting with dead hepatocytes, which would be a novel mechanism of NASH development.

## Results

### Cholesterol crystallization in hepatocytes and cholesterol loading to macrophages in NASH

**Figure S1.**
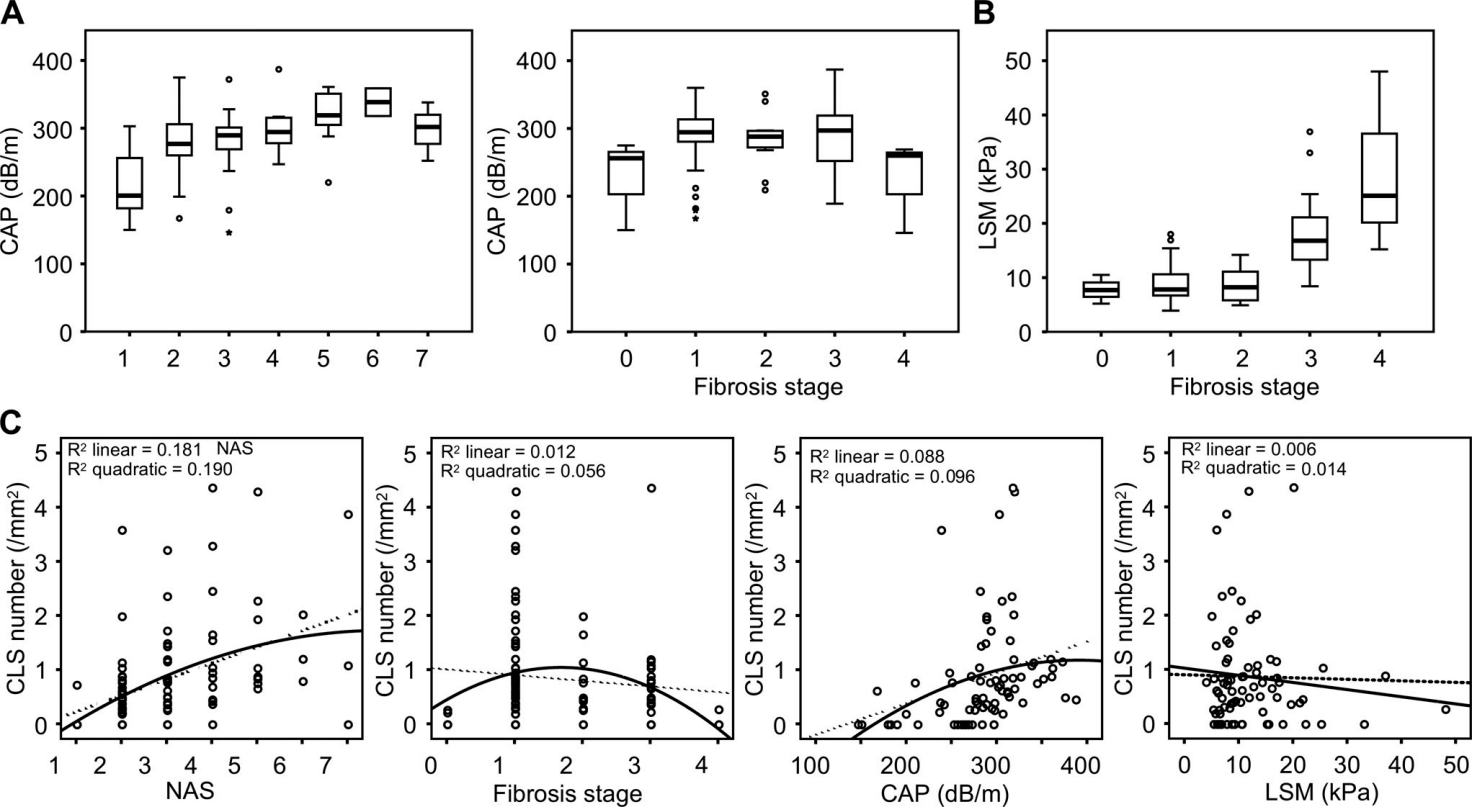
**Relationship between CLS number and clinical parameters in NAFLD/NASH subjects. (A)** Correlation of the CAP value measured by FibroScan, which represents hepatic lipid content, and histological scores of the livers (NAS and fibrosis stage). **(B)** Correlation of the LSM value measured by FibroScan and histological fibrosis stage. **(C)** Correlation of CLS number and NAS, fibrosis stage, CAP, and LSM values. Error bars represent means ± SEM.

Because CLS formation precedes the onset of liver fibrosis in a murine model of NASH, we compared the number of CLS with histological scores in liver biopsy specimens and measurements of FibroScan, a noninvasive imaging modality, in patients at an earlier stage of NAFLD/NASH. A total of 98 patients with mild liver dysfunction and hyperglycemia were analyzed (Table S1). The score of the controlled attenuation parameter (CAP), which represents hepatic lipid content, was increased in parallel with the NAFLD activity score (NAS) and decreased in patients with advanced fibrosis (Fig. S1 A). The score of liver stiffness measurement (LSM) was increased only in advanced fibrosis (fibrosis stage > 3; Fig. S1 B) as reported (Eddowes et al., 2019). The number of CLS was elevated at the early stages of NASH (corresponding to a NAS score of 5 and a fibrosis stage of 1 or 2), whereas that was rather decreased in the advanced stages of NASH (Fig. S1 C), consistent with our previous data (Itoh et al., 2013, 2017). These findings indicate that CLS is an earlier marker for liver fibrosis than LSM and raise a strong need to investigate the underlying mechanisms of CLS formation for better understanding the initial stage of NASH.

**Figure 1.**
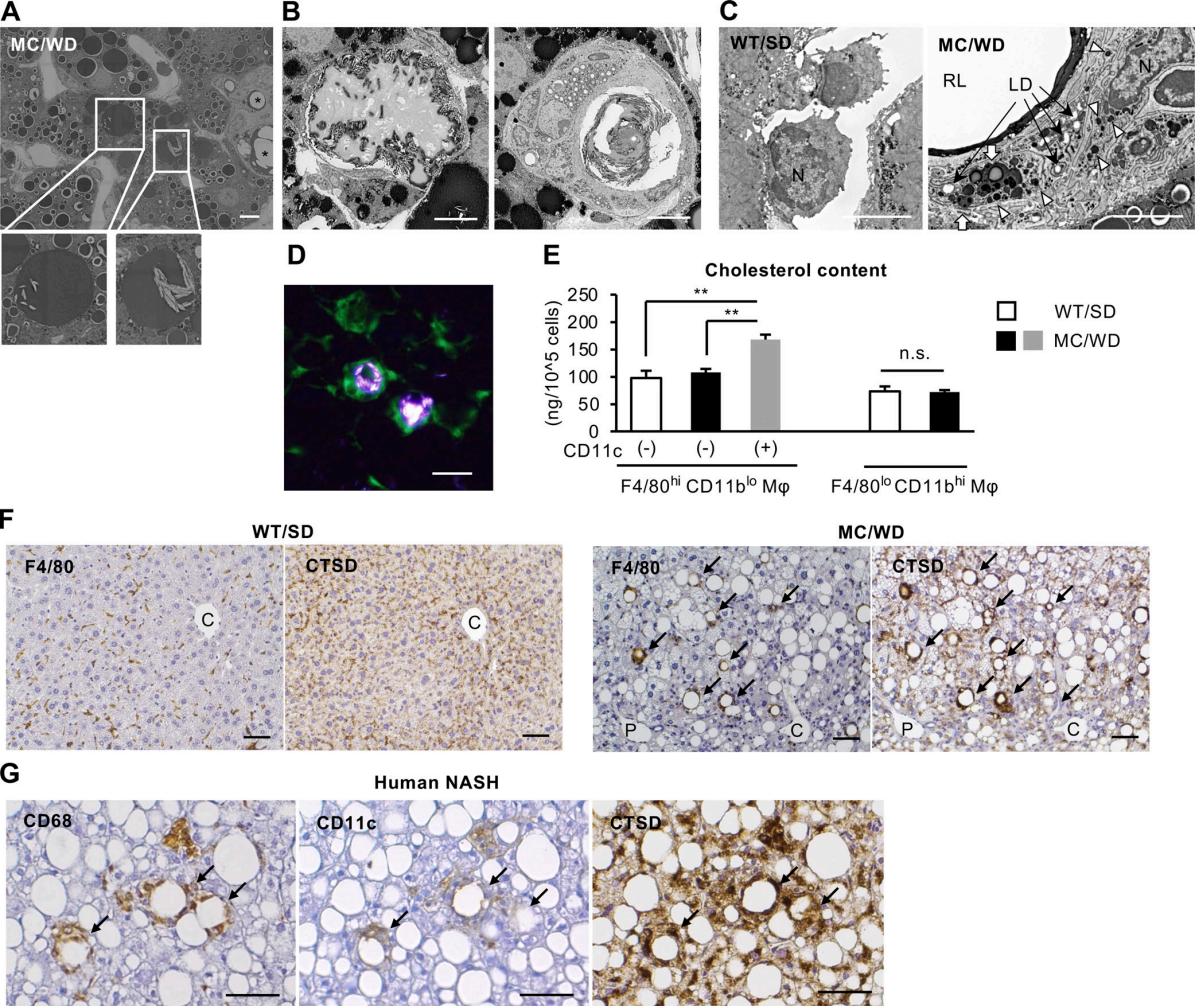
**Cholesterol crystallization and lysosomal stress in CLS-constituting macrophages in NASH livers. (A–C)** Electron micrographs of NASH livers from MC4R-KO mice fed a WD for 20 wk (MC/WD) and WT mice kept on an SD (WT/SD). **(A)** Fine cholesterol crystals were observed in lipid droplets of hepatocytes (insets). Asterisks, CLS. Scale bar, 10 μm. **(B)** Cholesterol crystallization in the remnant lipids of dead hepatocyte surrounded by macrophages (left), and lipid accumulation in CLS-constituting macrophages (right). Scale bars, 10 μm. **(C)** Macrophages in sinusoids of normal liver (left) and CLS-constituting macrophage (right). N, nucleus; LD, lipid droplets; RL, remnant lipid of dead hepatocyte; arrowheads, lysosomes; white arrows, autolysosomes. Scale bars, 5 μm. **(D)** Representative image of polarized light microscope of the liver from MC4R-KO mice transplanted with bone marrow cells from GFP-transgenic mice and fed a WD for 20 wk. Scale bar, 10 μm. **(E)** Total cholesterol content of macrophages isolated from normal (WT/SD) and NASH livers (MC/WD). Gating strategies for F4/80^hi^ CD11b^lo^ macrophages (Mφ): CD45^+^ Ly6G^−^ SiglecF^−^ F4/80^hi^ CD11b^lo^; F4/80^lo^ CD11b^hi^ Mφ: CD45^+^ Ly6G^−^ SiglecF^−^ F4/80^lo^ CD11b^hi^. F4/80^hi^ CD11b^lo^ macrophages were separated based on the expression levels of CD11c. *n* = 4. **P < 0.01; n.s., not significant. **(F)** Serial sections of the livers from WT mice fed an SD and MC4R-KO mice fed a WD stained with F4/80 and CTSD antibodies. Arrows, CLS; C, central veins; P, portal veins. Scale bars, 50 μm. **(G)** Serial sections of the livers from NASH patients stained with CD68, CD11c, and CTSD. Arrows, CLS. Scale bars, 50 μm. Data and images are representative of two independent experiments (A–F). Error bars represent means ± SEM.

Thus, we performed electron microscopic analysis of the livers of MC4R-KO mice fed a Western diet (WD) for 20 wk, which exhibited obesity, insulin resistance, and NASH-like liver phenotypes (Farrell et al., 2019; Itoh et al., 2011). Fine cholesterol crystals were observed within the lipid droplets of hepatocytes (Fig. 1 A). Cholesterol crystals were also present in the remnant lipids of dead hepatocytes surrounded by macrophages, and lipid accumulation was conversely observed in macrophages when the remnant lipids were reduced (Fig. 1 B). Unlike macrophages in the normal liver, numerous lipid droplets, lysosomes, and autolysosomes were observed in the cytoplasm of macrophages in NASH livers (Fig. 1 C). Polarized light microscopy also revealed the presence of large cholesterol crystals in CLS (Fig. 1 D). Cellular cholesterol content was increased only in the CD11c-positive F4/80^hi^ CD11b^lo^ macrophages from NASH livers, which form CLS (Itoh et al., 2017), compared with the CD11c-negative macrophages from normal and NASH livers (Fig. 1 E). On the other hand, cholesterol content remained unchanged in F4/80^lo^ CD11b^hi^ macrophages (Fig. 1 E). In atherosclerotic plaques, cholesterol taken up by macrophages undergoes degradation in lysosomes and induces lysosomal stress (Sergin et al., 2015). Similarly, the enhanced immunostaining of lysosomal enzyme cathepsin D (CTSD) was observed in the macrophages forming CLS in MC4R-KO mice and human NASH (Fig. 1, F and G). These data suggest that remnant lipids, including cholesterol crystals, in dead hepatocytes induce lysosomal stress in CLS-constituting macrophages both in mice and humans.

### Optimization of chemically modified βCD-PRX targeting the liver

**Figure 2.**
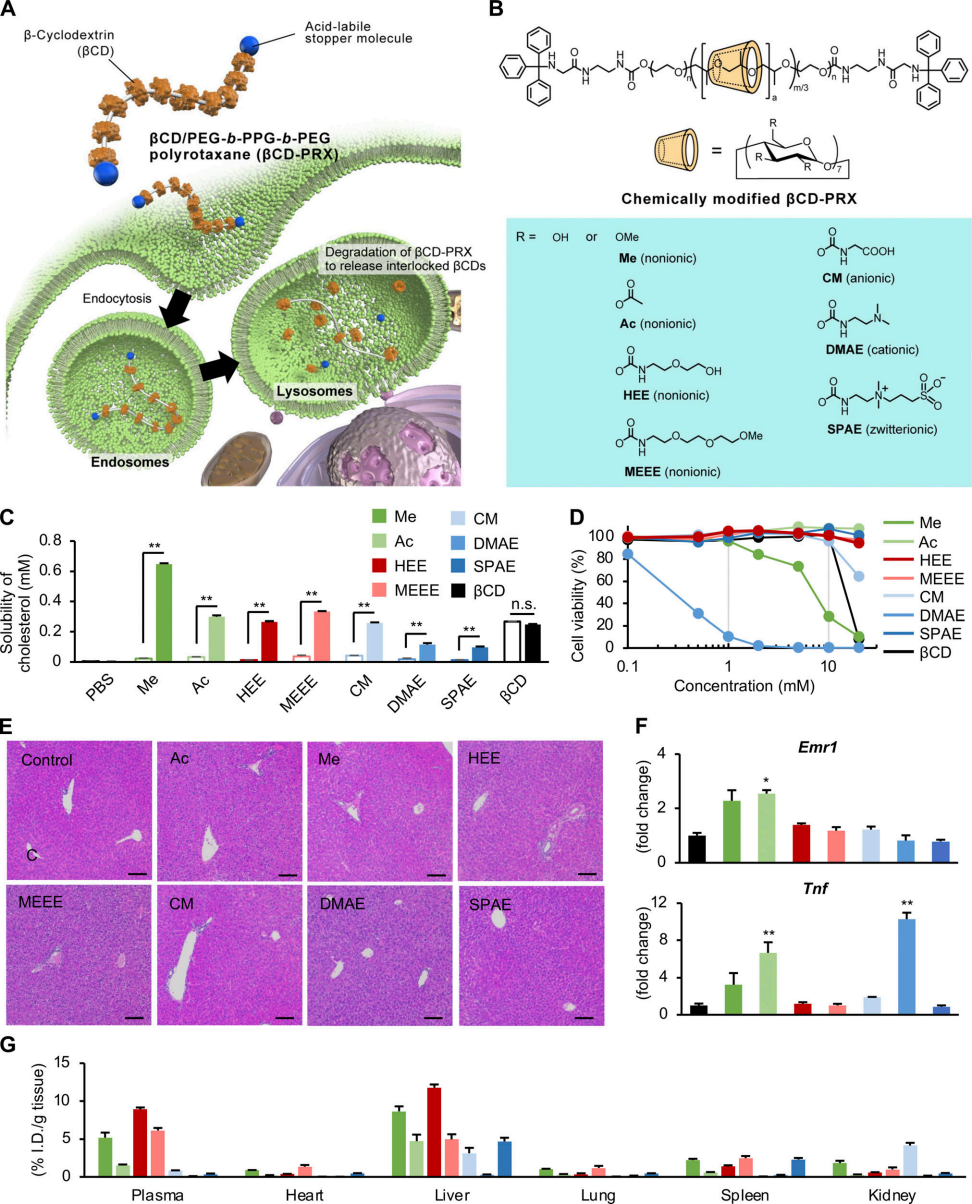
**Optimization of chemical modification for βCD-PRXs. (A)** Schematic illustration showing the mechanism of action of βCD-PRX. **(B)** Structure of chemically modified βCD-PRXs. Me, methyl; Ac, acetyl; MEEE, 2-(2-(2-methoxyethoxy)ethoxy)ethyl carbamate; CM, carboxymethyl carbamate; and SPAE, 2-(*N*-3-sulfopropyl-*N*,*N*-dimethylammonium)ethyl carbamate. **(C)** Cholesterol binding capacity evaluated by the solubility of cholesterol in the presence of each βCD-PRX and HP-βCD (βCD) under neutral and acidic pH conditions. Open bar, pH 7.4; solid bar, pH 5.0. *n* = 3. **P < 0.01, n.s.; not significant. **(D)** Cytotoxicity of chemically modified βCD-PRXs and βCD in RAW264 macrophages. *n* = 5. **(E)** Representative images of hematoxylin and eosin staining of the livers from WT mice treated with chemically modified βCD-PRXs at a dose of 200 mg/kg for 24 h. **(F and G)** Hepatic mRNA expression of inflammatory genes (F) and tissue distribution evaluated by fluorescence intensities (G) in WT mice 24 h after subcutaneous injection of Cy5.5-labeled βCD-PRXs at a dose of 200 mg/kg. *Emr1*, EGF-like module-containing mucin-like hormone receptor-like 1 (F4/80); *TNF*, tumor necrosis factor-α. *n* = 5. *P < 0.05, **P < 0.01 versus PBS. Data and images are representative of two independent experiments. Error bars represent means ± SEM.

To elucidate the role of lysosomal cholesterol overload in CLS-constituting macrophages, we came up with a unique supramolecule, βCD-PRX (Tamura et al., 2016; Tamura and Yui, 2018). 2-Hydroxypropyl-βCD (HP-βCD) is known to form the inclusion complex with free cholesterol and remove it from the cells (Kilsdonk et al., 1995). βCD-threaded acid-degradable PRXs are preferentially distributed to endosomes and lysosomes through endocytosis, and the stopper molecules in βCD-PRXs are designed to release βCD under lysosomal acidic conditions (Fig. 2 A). Thus, βCD-PRXs serve as carriers to deliver βCD to lysosomes efficiently. Our group has developed various chemically modified βCD-PRXs because the chemical modification plays an essential role in imparting solubility in aqueous media and modulating body disposition (Nishida et al., 2016; Ohashi et al., 2021; Tamura et al., 2022; Tamura and Yui, 2014, 2015; Tonegawa et al., 2020; Zhang et al., 2021).

To identify the optimal chemical modification for liver targeting, seven series of βCD-PRXs with different chemical modifications, including nonionic, anionic, cationic, and zwitterionic groups were synthesized and tested (Fig. 2 B and Table S2). When the inclusion ability of cholesterol was tested at pH 7.4, all chemically modified βCD-PRXs showed negligible ability to solubilize free cholesterol, unlike HP-βCD (Fig. 2 C), because the inclusion of cholesterol was inhibited by the occupation of the hydrophobic cavity of threaded βCDs with an axial polymer chain. In contrast, all chemically modified PRXs solubilized cholesterol at pH 5.0 because the released βCDs formed an inclusion complex with free cholesterol (Fig. 2 C). Cytotoxicity tests revealed cytotoxicity of several chemically modified βCD-PRXs at high concentrations (methyl, carboxymethyl carbamate, and 2-(*N*,*N*-dimethylamino)ethyl carbamate [DMAE]), and HP-βCD (Fig. 2 D). We also examined the toxicity of various chemically modified βCD-PRX in vivo 24 h after subcutaneous administration into WT mice. Serum levels of aspartate aminotransferase, alanine aminotransferase (ALT), and alkaline phosphatase were elevated in mice treated with DMAE-modified βCD-PRX (Table S3). Histological examinations revealed immune cell infiltration into the livers in DMAE-modified βCD-PRX, and hepatic expression of inflammatory genes was upregulated in methyl, acetyl, and DMAE-modification groups consistent with the cytotoxicity test (Fig. 2, E and F). Among others, 2-(2-hydroxyethoxy)ethyl carbamate (HEE)–modified βCD-PRX showed only minimal effects on these parameters. A biodistribution study of chemically modified βCD-PRXs reveled that HEE modification demonstrated the highest accumulation in the liver in WT mice (Fig. 2 G). Thus, the HEE group-modified βCD-PRX (hereafter indicated as βCD-PRX) was used in the subsequent studies.

**Figure 3.**
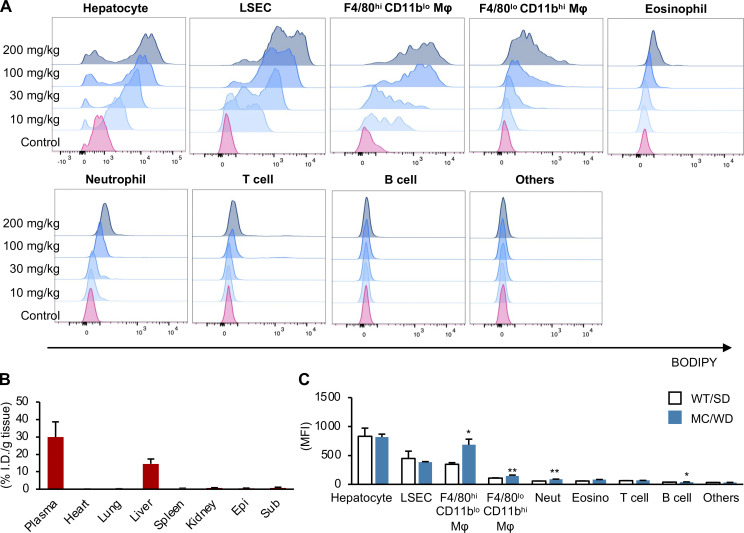
**Biodistribution of βCD-PRX in the liver. (A)** Cellular uptake of BODIPY-modified HEE-βCD-PRX at various dosages in WT mice. **(B)** Tissue distribution of HEE-βCD-PRX evaluated by fluorescence intensities in MC4R-KO mice fed a WD for 20 wk. *n* = 3. **(C)** Comparison of HEE-βCD-PRX distribution at a dose of 200 mg/kg in WT mice fed an SD (WT/SD) and MC4R-KO mice fed a WD for 20 wk (MC/WD). *n* = 3. *P < 0.05, **P < 0.01 versus WT/SD. Data are representative of two independent experiments. Error bars represent means ± SEM. MFI, mean fluorescence intensity.

We examined cellular distribution of BODIPY-labeled βCD-PRX in the livers of WT mice treated with a single injection of βCD-PRX at different doses. βCD-PRX uptake was observed mainly in hepatocytes, sinusoidal endothelial cells, and macrophages in a dose-dependent manner, whereas little uptake was detected in the (CD45 and CD146-negative) “others” fraction, including fibroblasts and biliary epithelial cells (Fig. 3 A). Moreover, we confirmed a hepatotrophic biodistribution in a NASH model (MC4R-KO mice fed a WD for 20 wk; Fig. 3 B). The uptake rate was significantly increased in several cell types after the onset of NASH, but not in the other fractions (Fig. 3 C). Taken together, we considered that the HEE group is the optimal chemical modification for further study.

### βCD-PRX attenuates liver fibrosis by reducing cholesterol accumulation in macrophages

**Figure 4.**
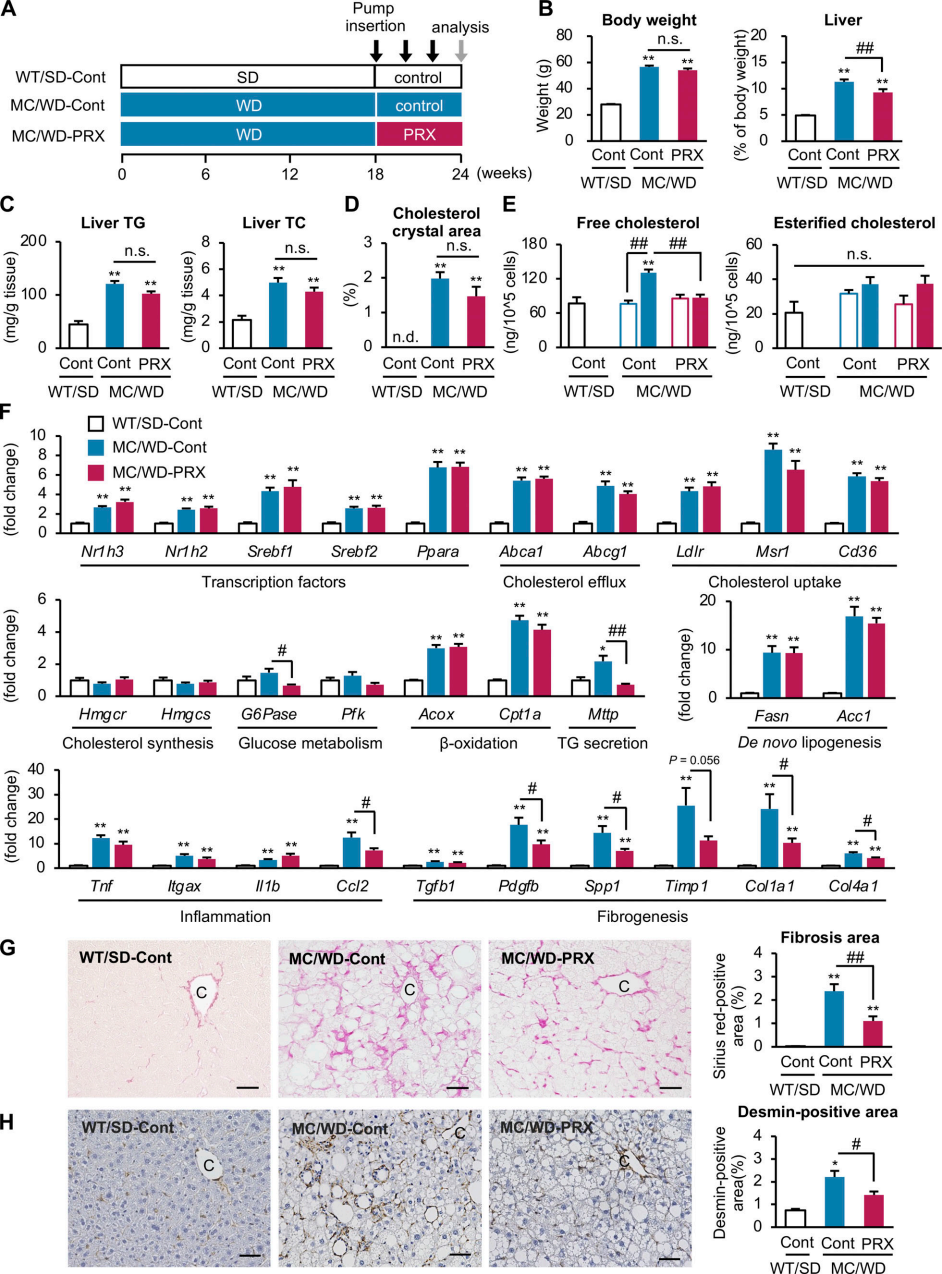
**Effect of βCD-PRX on liver fibrosis in a mouse model of NASH. (A)** Experimental protocol of βCD-PRX treatment in a NASH model using MC4R-KO mice. After the development of NASH with 18-wk WD feeding, MC4R-KO mice were received implantation of osmotic minipumps at a dose of 30 mg/kg/d of βCD-PRX or normal saline as a control (Cont) for an additional 6 wk. PRX, βCD-PRX. WT/SD-cont, *n* = 6; MC/WD-cont, *n* = 9; MC/WD-PRX, *n* = 8. **(B)** Body weight and liver weight after βCD-PRX treatment. **(C)** Hepatic TG and total cholesterol (TC) content. **(D)** Area of cholesterol crystals in the liver evaluated by polarized light microscope. **(E)** Free and esterified cholesterol content of F4/80^hi^ CD11b^lo^ macrophages isolated from the livers at the end of the experiment. Open bars, CD11c-negative macrophages; closed bars, CD11c-positive macrophages. **(F)** Hepatic mRNA expression of genes related to lipid metabolism, inflammation, and fibrogenesis. *Nr1h3*, nuclear receptor subfamily 1 group H member 3 (LXRα); *Nr1h2*, nuclear receptor subfamily 1 group H member 2 (LXRβ); *Srebf*, sterol regulatory element binding transcription factor; *Ppara*, peroxisomal proliferator-activated receptor α; *Abca1*, ATP binding cassette subfamily A member 1; *Agcg1*, ATP biding cassette subfamily G member 1; *Ldlr*, low density lipoprotein receptor; *Msr1*, macrophage scavenger receptor 1; *Hmgcr*, hydroxymethylglutaryl-CoA reductase; *Hmgcs*, hydroxymethylglutaryl-CoA synthase; *G6pase,* glucose 6-phosphatase; *Pfk,* 6-phosphofructokinase; *Acox*, peroxisomal acyl-coenzyme A oxidase 1; *Cpt1a*; carnitine palmitoyltransferase 1A; *Mttp*, microsomal triglyceride transfer protein; *Fasn*, fatty acid synthase; *Acc1*, acetyl-CoA carboxylase 1; *Itgax*, integrin subunit αX (CD11c); *IL1β*, interleukin-1β; *Ccl2*, C-C motif chemokine ligand 2; *Tgfβ1*, transforming growth factor β1; *Pdgfb*, platelet-derived growth factor subunit B; *Spp1*, secreted phosphoprotein 1; *Timp1*, tissue inhibitor of metalloproteinase 1; *Col1a1*, collagen type I α chain; and *Col4a1*, collagen type IV α chain. **(G and H)** Fibrosis area evaluated by Sirius red staining (G) and quantification of desmin-positive area (H). C, central veins. Scale bars, 50 μm. *P < 0.05, **P < 0.01 versus WT/SD-Cont; ^#^P < 0.05, ^##^P < 0.01. Data and images are representative of two independent experiments. Error bars represent means ± SEM.

**Figure S2.**
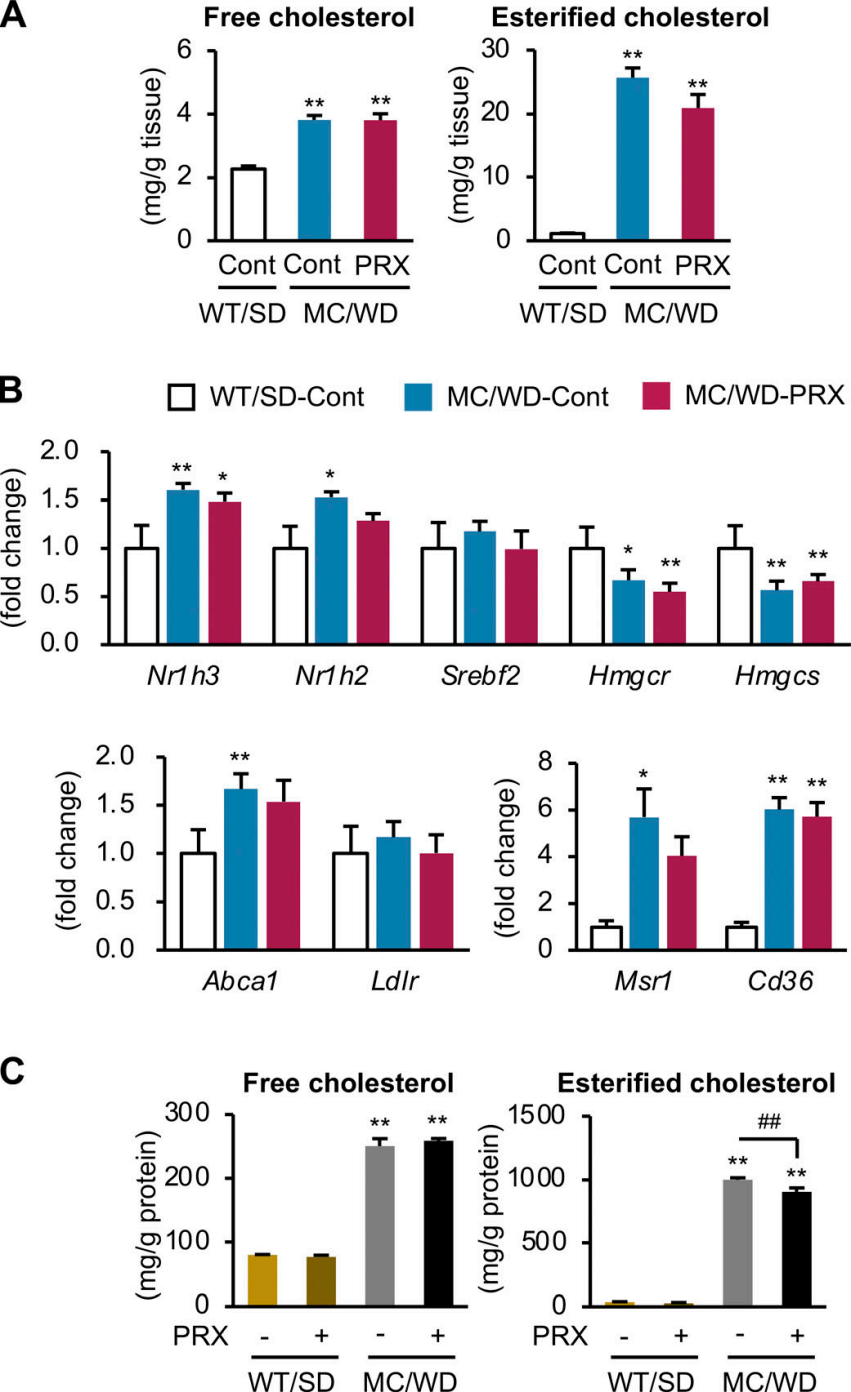
**Effect of βCD-PRX on cholesterol accumulation in hepatocytes. (A)** Free and esterified cholesterol content of the livers from WT mice and MC4R-KO mice fed a WD for 24 wk. Cont, control. WT/SD-Cont, *n* = 6; MC/WD-Cont, *n* = 9; MC/WD-PRX, *n* = 8. **P < 0.01 versus WT/SD-Cont. **(B)** mRNA expression of genes related to cholesterol metabolism in hepatocytes isolated from WT mice fed an SD and MC4R-KO mice fed a WD for 4 wk, and treated with βCD-PRX for the last 1 wk. *n* = 5. *P < 0.05, **P < 0.01 versus WT/SD-Cont. **(C)** Free and esterified cholesterol content of primary hepatocytes treated with βCD-PRX for 24 h. Primary hepatocytes were isolated from WT mice fed an SD and MC4R-KO mice fed a WD for 10 d, and treated with βCD-PRX for 24 h *n* = 3. **P < 0.01 versus WT/SD-Veh; ^##^P < 0.01. Data are representative of two (A and B) or three (C) independent experiments. Error bars represent means ± SEM.

To elucidate the pathophysiological role of lysosomal cholesterol accumulation in CLS-constituting macrophages in the development of NASH, MC4R-KO mice received subcutaneous administration of βCD-PRX for 6 wk, after they developed NASH (Fig. 4 A). The liver weight and serum ALT levels were decreased with βCD-PRX treatment, whereas the serum and hepatic levels of triglyceride and (total, free, and esterified) cholesterol and the area of cholesterol crystals in the liver did not change (Fig. 4, B–D; Fig. S2 A; and Table 1). Using isolated hepatocytes from MC4R-KO mice treated with βCD-PRX, we found that βCD-PRX did not affect mRNA expression of lipid metabolism–related genes in hepatocytes (Fig. S2 B). βCD-PRX also showed only marginal effects on cholesterol contents in primary cultured hepatocytes prepared from normal and steatotic livers (Fig. S2 C). In contrast, βCD-PRX treatment markedly inhibited the increase in the free cholesterol content of the CD11c-positive macrophages (localizing to CLS) from NASH livers (P < 0.01, Fig. 4 E). Moreover, βCD-PRX treatment markedly suppressed hepatic mRNA expression of *Ccl2* and profibrotic factors, along with their serum cytokine levels, in the NASH model (Fig. 4 F and Table S4) without remarkable impact on hepatic expression of genes related to cholesterol metabolism, de novo lipogenesis, β-oxidation, and glucose metabolism, except *Mttp*, which mediates very-low-density lipoprotein secretion from the livers (Fig. 4 F). Moreover, Sirius red staining and desmin immunostaining (representing activated fibroblasts) revealed that βCD-PRX effectively suppressed liver fibrosis (Fig. 4, G and H). These observations, taken together, indicate that βCD-PRX ameliorates the development of liver fibrosis by mainly acting on macrophages rather than hepatocytes and fibroblasts.

**Table 1.** Serological parameters of MC4R-KO and WT mice treated with βCD-PRX for 6 wk

	WT/SD	MC/WD	
	Cont	Cont	PRX
TG (mg/dl)	112.5 ± 1.8	120.1 ± 8.8	134.4 ± 16.5
TC (mg/dl)	93.7 ±4.4	351.3 ± 20.0**	317.3 ± 18.2**
ALT (U/liter)	35.8 ± 1.8	287.9 ± 38.3**	173.9 ± 34.1**^#^
Insulin (ng/ml)	0.4 ± 0.1	33.6 ± 11.9*	23.0 ± 7.9

MC, MC4R-KO mice; Cont, control; PRX, βCD-PRX; TC, total cholesterol. WT/SD-Cont, *n* = 6; MC/WD-Cont, *n* = 9; MC/WD-PRX, *n* = 8. **P < 0.01 versus WT/SD-Cont; ^#^P < 0.05 versus MC/WD-Cont. Data are representative of two independent experiments. Data are expressed as the mean ± SEM.

### Effect of βCD-PRX on lysosomal stress in CLS-constituting macrophages

**Figure 5.**
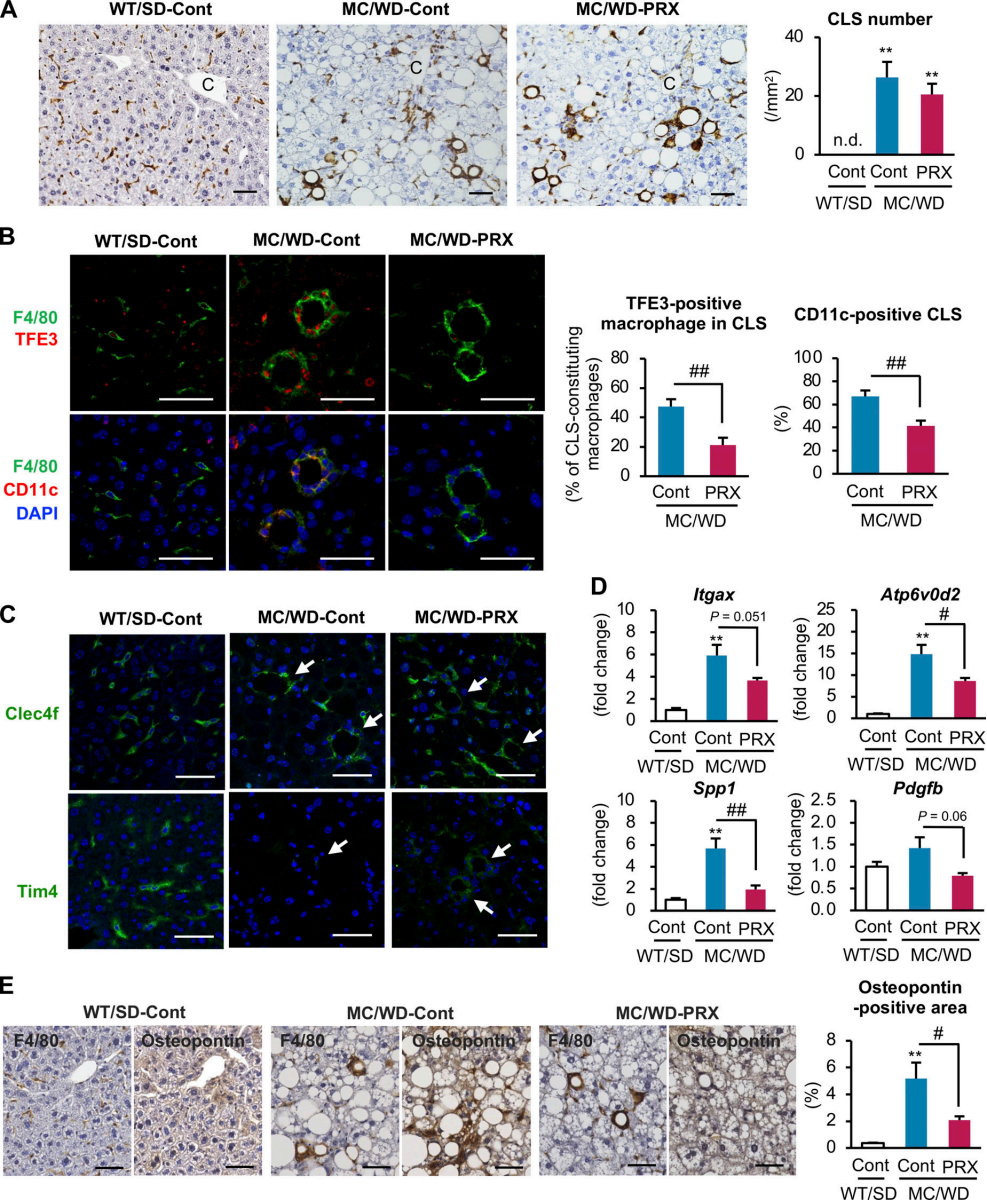
**Effect of βCD-PRX on inflammatory and profibrotic phenotypes of CLS-constituting macrophages in NASH.** Immunohistochemistry of the livers of MC4R-KO mice treated with βCD-PRX for 6 wk. **(A)** F4/80 immunostaining and quantification of CLS number. C, central veins. Scale bars, 50 μm. WT/SD-Cont, *n* = 6; MC/WD-Cont, *n* = 9; MC/WD-PRX, *n* = 8. **(B)** Immunofluorescence staining of TFE3, F4/80, and CD11c, and quantification of the rates of TFE3 nuclear translocation and CD11c-positive CLS. Nuclei were counterstained with DAPI. Scale bars, 50 μm. MC/WD-Cont, *n* = 9; MC/WD-PRX, *n* = 8. **P < 0.01 versus MC/WD-Cont. **(C)** Representative images of Clec4f and Tim4 immunostaining of the livers from WT and MC4R-KO mice. Arrows, CLS. Scale bars, 50 μm. **(D)** mRNA expression levels of genes related to NASH-specific macrophage phenotypes, including *Itgax*, *Atp6v0d2*, *Spp1*, and *Pdgfb* in F4/80^hi^ CD11b^lo^ macrophages sorted from livers of MC4R-KO mice after 6-wk βCD-PRX treatment. *n* = 3. **(E)** Representative images of serial sections stained with anti-F4/80 and Osteopontin antibodies. Scale bars, 50 μm. WT/SD-Cont, *n* = 6; MC/WD-Cont, *n* = 9; MC/WD-PRX, *n* = 8. **P < 0.01 versus WT/SD-Cont. ^#^P < 0.05, ^##^P < 0.05. Data and images are representative of two independent experiments. Error bars represent means ± SEM.

Next, we evaluated the effect of βCD-PRX on the macrophages constituting CLS because these macrophages are supposed to interact with dead hepatocytes containing cholesterol crystals. F4/80 immunostaining revealed the comparable formation of CLS, regardless of βCD-PRX treatment (Fig. 5 A). We have previously identified increased lysosomal stress and subsequent activation of transcription factor E3 (TFE3) in the CLS-constituting macrophages by which CD11c expression was induced in these cells (Kanamori et al., 2021). In this study, nuclear TFE3 immunostaining in CLS was markedly suppressed by βCD-PRX treatment, along with CD11c expression (Fig. 5 B). These CLS-constituting macrophages were positive for C-type lectin domain family 4 member F (Clec4f) immunostaining in our NASH model as previously reported (Itoh et al., 2017). Interestingly, TIM4 immunostaining was almost absent in the macrophages forming the CLS in the control group. In contrast, there was a modest positive signal observed in the βCD-PRX–treated group, suggesting that βCD-PRX treatment affects the gene expression profiles related to Kupffer cell (KC) identity (Fig. 5 C). βCD-PRX treatment also suppressed gene expression of *Atp6v0d2* (a lysosomal stress marker), *Itgax* (CD11c), profibrotic *Spp1* (osteopontin), and *Pdgfb* in F4/80^hi^ CD11b^lo^ macrophages isolated from the livers at the end of the experiment (Fig. 5 D). Histological analysis revealed osteopontin immunostaining in CLS and the osteopontin-positive area was reduced with βCD-PRX treatment in parallel with mRNA expression levels (Fig. 5 E).

**Figure S3.**
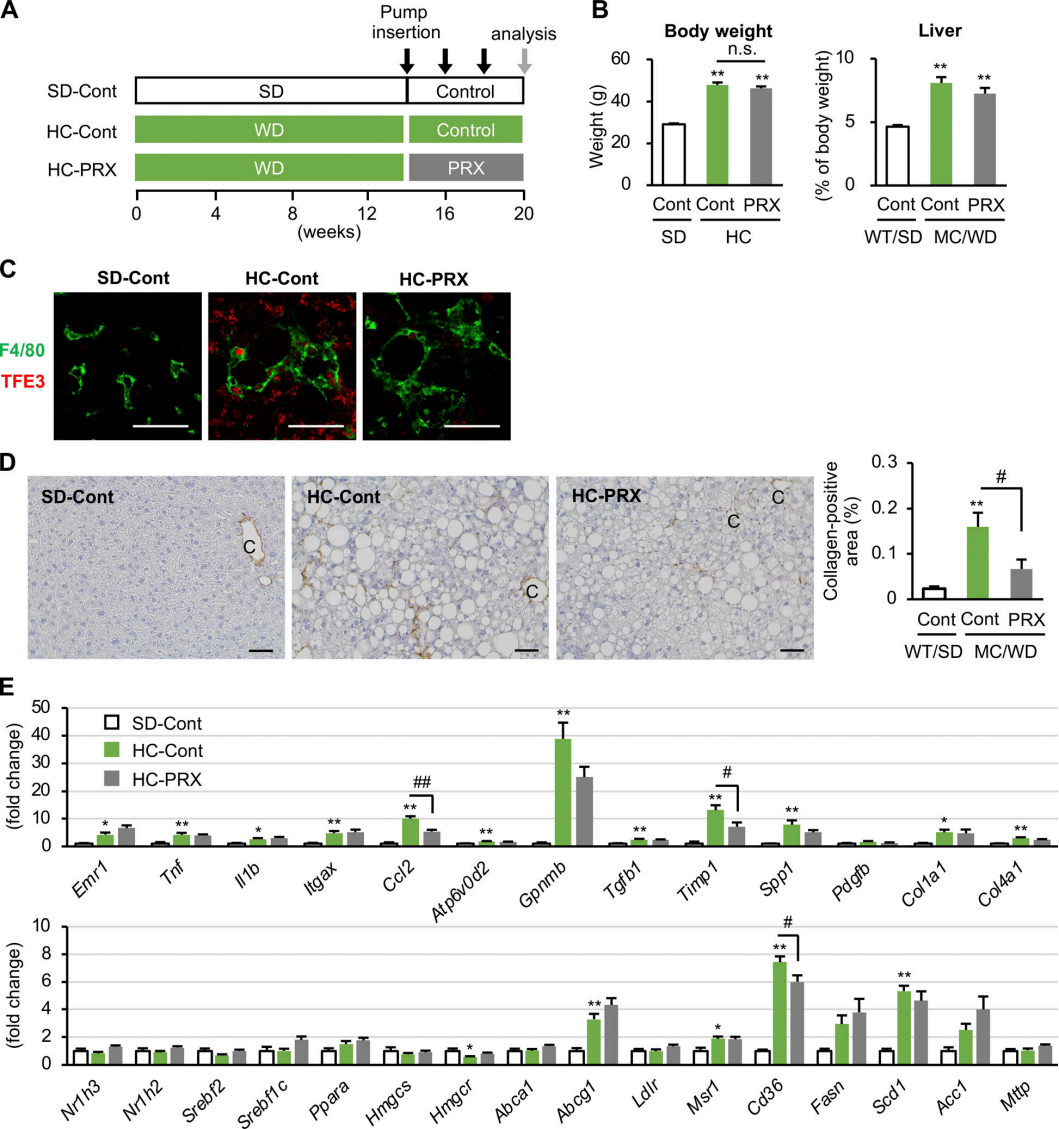
**Effect of βCD-PRX on liver fibrosis in a mouse model of NASH with high-fat and HC diet. (A)** Experimental protocol of βCD-PRX treatment in a NASH model with an HC diet for 20 wk. βCD-PRX was subcutaneously administered by osmotic minipumps at a dose of 30 mg/kg/d of βCD-PRX or normal saline as a control (Cont) for the last 6 wk. PRX, βCD-PRX. SD-Cont, *n* = 6; HC-Cont, *n* = 7; HC-PRX, *n* = 7. **(B)** Body weight and liver weight after βCD-PRX treatment. **(C)** Immunofluorescence staining of TFE3 and F4/80. Scale bars, 50 μm. **(D)** Fibrosis area evaluated by type III collagen immunostaining. C, central veins. Scale bars, 50 μm. **(E)** Hepatic mRNA expression of genes related to inflammation, fibrogenesis, and lipid metabolism. *P < 0.05, **P < 0.01 versus SD-cont; ^#^P < 0.05, ^##^P < 0.01. Error bars represent means ± SEM.

Moreover, we confirmed the effect of βCD-PRX in another mouse model of NASH, in which WT mice were fed a high-cholesterol (HC) diet for 20 wk (Boland et al., 2019), and received βCD-PRX treatment (30 mg/kg/d) during the last 6 wk (Fig. S3 A). There were no significant changes in body weight, liver weight, and liver triglyceride and cholesterol contents (Fig. S3 B and Table S5). βCD-PRX suppressed TFE3 nuclear translocation in CLS and fibrotic changes in the liver (Fig. S3, C–E). These observations support the therapeutic effect of βCD-PRX on NASH development independently of MC4R signaling. Collectively, these observations indicate that βCD-PRX treatment ameliorates lysosomal stress response and suppresses profibrotic phenotypes of the CLS-constituting macrophages.

### Cholesterol crystals induce inflammatory and profibrotic changes in macrophages from steatotic livers

**Figure 6.**
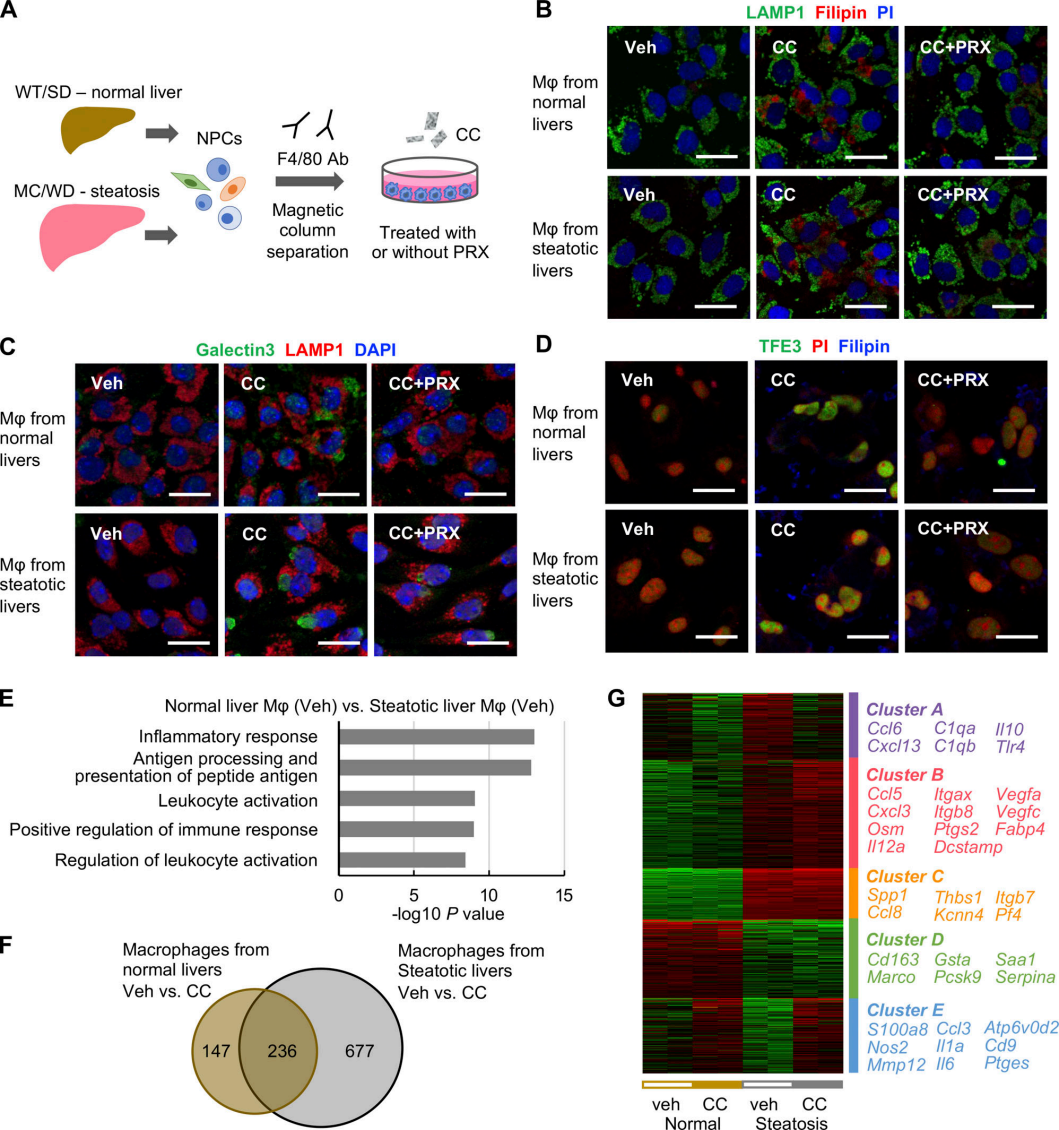
**Effect of cholesterol crystals and βCD-PRX on gene expression profiles in macrophages isolated from normal and steatotic livers. (A)** Experimental protocol using primary hepatic macrophages. Hepatic macrophages were isolated using magnetic columns from normal livers (WT mice fed an SD) and steatotic livers (MC4R-KO mice fed a WD for 6–10 wk), and were stimulated with cholesterol crystals (CC, 500 μg/ml) and βCD-PRX (1 mM) for 24 h. **(B)** Representative images of immunostaining of macrophages from normal livers and steatotic livers treated with CC and βCD-PRX for 24 h. Fixed cells were stained with LAMP1 (a lysosome marker, green), filipin (free cholesterol, red), and PI (propidium iodide, nuclei, blue). Scale bars, 20 μm. **(C and D)** Immunostaining of Galectin 3 (a marker of lysosomal membrane damage, green), LAMP1 (red), and DAPI (blue; C) and TFE3 (green), Filipin (blue), and PI (red; D) in hepatic macrophages. Scale bars, 20 μm. Images are representative of two independent experiments. **(E)** RNA-seq was conducted using hepatic macrophages isolated from normal livers and steatotic livers (*n* = 2). Gene ontology analysis of the genes twofold upregulated in hepatic macrophages isolated from steatotic livers compared with those from normal livers using Metascape. **(F)** Venn diagram showing the twofold upregulated genes in CC-treated macrophages compared to each Veh. **(G)** Unsupervised hierarchical clustering analysis using RNA-seq data (*k* = 5). Genes belonging to each cluster are indicated on the right.

**Figure 7.**
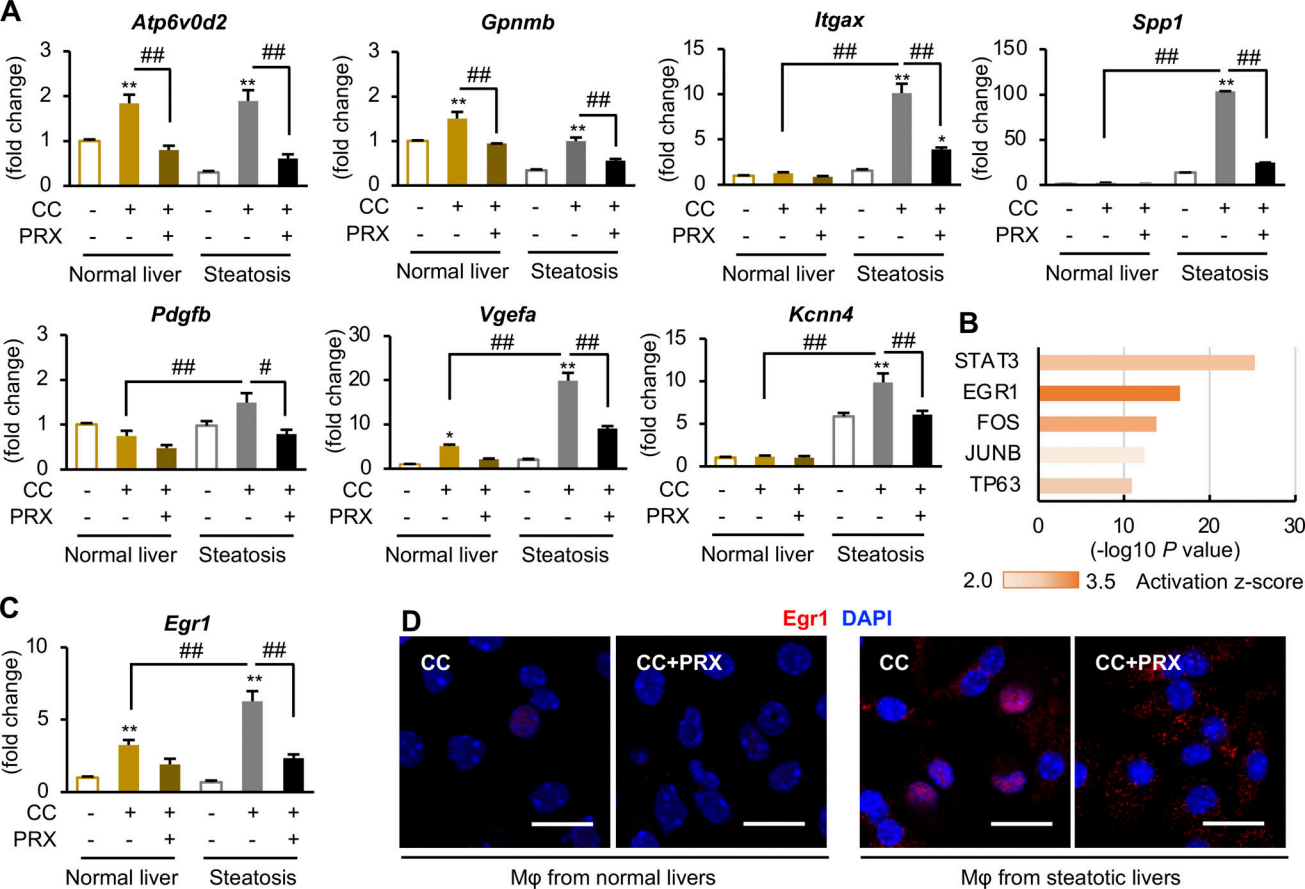
**Differential response to cholesterol crystal (CC)–induced lysosomal stress in macrophages isolated from normal and steatotic livers. (A)** mRNA expression levels analyzed by quantitative real-time PCR in hepatic macrophages. *Gpnmb*, glycoprotein nonmetastatic melanoma protein B; *Vegfa*, vascular endothelial growth factor A); *Kcnn4*, potassium calcium–activated channel, subfamily N, member 4. **(B)** Top five upstream transcription regulators specific for CC-treated macrophages from steatotic livers analyzed by ingenuity pathway analysis. **(C)** *Egr1* expression in hepatic macrophages. *n* = 4. *P < 0.05, **P < 0.01 versus each Veh; ^##^P < 0.01. **(D)** Immunostaining of Egr1 (red) in hepatic macrophages (Mφ). Scale bars, 20 μm. Data and images are representative of two independent experiments.

We next performed cholesterol loading experiments in vitro using hepatic macrophages prepared from WT mice on a standard diet (SD; normal livers) and MC4R-KO mice fed a WD for 6–8 wk (steatotic livers; Fig. 6 A). Cholesterol crystals were added to hepatic macrophages, and then lysosomes and cholesterol were stained with lysosome-associated membrane protein (LAMP) 1 and Filipin, respectively (Fig. 6 B). Treatment with cholesterol crystals resulted in increased expression of galectin-3, a marker of lysosomal membrane damage, and nuclear translocation of TFE3. These effects were reversed by βCD-PRX treatment (Fig. 6, C and D). RNA sequencing (RNA-seq) was conducted to compare gene expression profiles between hepatic macrophages from normal livers and steatotic livers treated with cholesterol crystals (*n* = 2). Gene ontology analysis of 1,049 genes upregulated (>twofold) in macrophages from steatotic livers relative to those from normal livers revealed activation of inflammatory pathways in steatotic livers (Fig. 6 E). The number of genes, whose expression was increased by the treatment with cholesterol crystals, was much higher in macrophages from steatotic livers (Fig. 6 F). Unsupervised hierarchical clustering revealed differential responses to cholesterol crystals between the macrophages from normal and steatotic livers (Fig. 6 G). Several inflammatory cytokines and lysosome-related genes (*Atp6v0d2*) were similarly upregulated by the treatment with cholesterol crystals in the macrophages from normal and steatotic livers (cluster E), whereas genes characteristic for scar-associated macrophage (SAM) or NASH-associated macrophage (Ramachandran et al., 2019; Xiong et al., 2019), such as *Itgax*, *Vegfa*, *Fabp4*, *Spp1*, and *Kcnn4*, were upregulated only in the macrophages from steatotic livers (clusters B and C). We confirmed the data by quantitative real-time PCR (Fig. 7 A). Intriguingly, ingenuity pathway analysis using RNA-seq data revealed early growth response 1 (Egr1) activation as an upstream transcription regulator specific for steatotic liver–derived hepatic macrophages treated with cholesterol crystals (Fig. 7 B). We also found increased mRNA expression and nuclear translocation of Egr1 in hepatic macrophages isolated from steatotic livers (Fig. 7, C and D). It has been reported that Egr1 induces production of adhesion molecules, cytokines, and growth factors including osteopontin and platelet-derived growth factor (PDGF) in certain cell types (Khachigian and Collins, 1997; Wang et al., 2021; Wu et al., 2009). These data indicate that cholesterol crystals induce profibrotic changes only in macrophages from steatotic livers, which may acquire proinflammatory traits after exposure to the microenvironmental milieu of steatosis.

### βCD-PRX alleviates cholesterol crystal–induced lysosomal dysfunction by promoting cholesterol excretion

**Figure 8.**
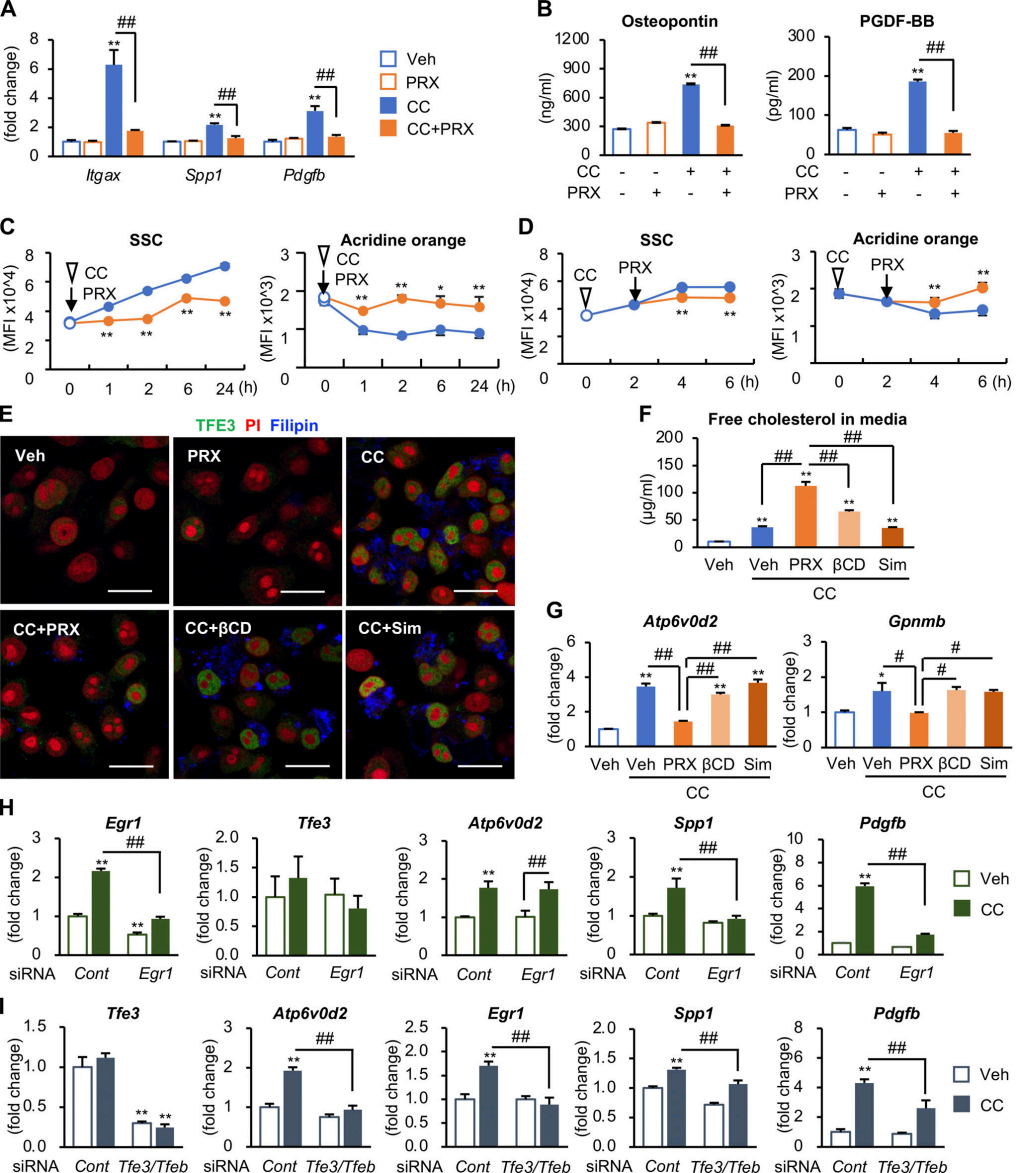
**Effect of βCD-PRX on lysosomal dysfunction and profibrotic changes in RAW264 macrophages. (A)** mRNA expression of genes related to NASH-specific macrophage phenotypes after incubation with cholesterol crystal (CC) and βCD-PRX for 24 h. **P < 0.01 versus Veh; ^##^P < 0.01. **(B)** Osteopontin and PDGF-BB secretion from RAW264 macrophages in response to CC and βCD-PRX. **(C and D)** Evaluation of lysosomal function using acridine orange in RAW264 macrophages. Side scatter (SSC) intensity represents uptake of CCs, and fluorescence of acridine orange was detected by PerCP channel. βCD-PRX was added at the same time with CC (C) or 2 h after CC treatment (D). Blue line, CC; orange line, CC + βCD-PRX. *n* = 4. *P < 0.05, **P < 0.01 versus CC at each timing. **(E)** Immunocytochemistry of RAW264 macrophages treated with CC in the presence of βCD-PRX (1 mM), HP-βCD (unthreaded in linear polymer; βCD, 1 mM), and Simvastatin (Sim, 1 μM) for 24 h. TFE3 (green), Filipin (blue), and PI (red). **(F and G)** Free cholesterol concentrations in the culture media (F) and mRNA expression of genes related to lysosomal stress in RAW264 treated with CC and βCD-PRX, βCD, and Sim (G). *n* = 4. *P < 0.05, **P < 0.01 versus Veh; ^#^P < 0.05, ^##^P < 0.01. **(H)** The effect of Egr1 knockdown on expression of *Spp1* and *Pdgfb*. Macrophages were transfected with siRNA targeting Egr1 and negative control, and treated with CC for 6 h. **(I)** Role of TFE3 and TFEB transcription factors in CC-induced *Egr1* expression in RAW264 macrophages. Macrophages were transfected with siRNA targeting Tfe3/Tfeb and negative control, and treated with CC for 6 h *n* = 4. **P < 0.01 versus Cont-Veh; ^##^P < 0.01. Data and images are representative of three (A–G) or two (H and I) independent experiments. Error bars represent means ± SEM.

**Figure S4.**
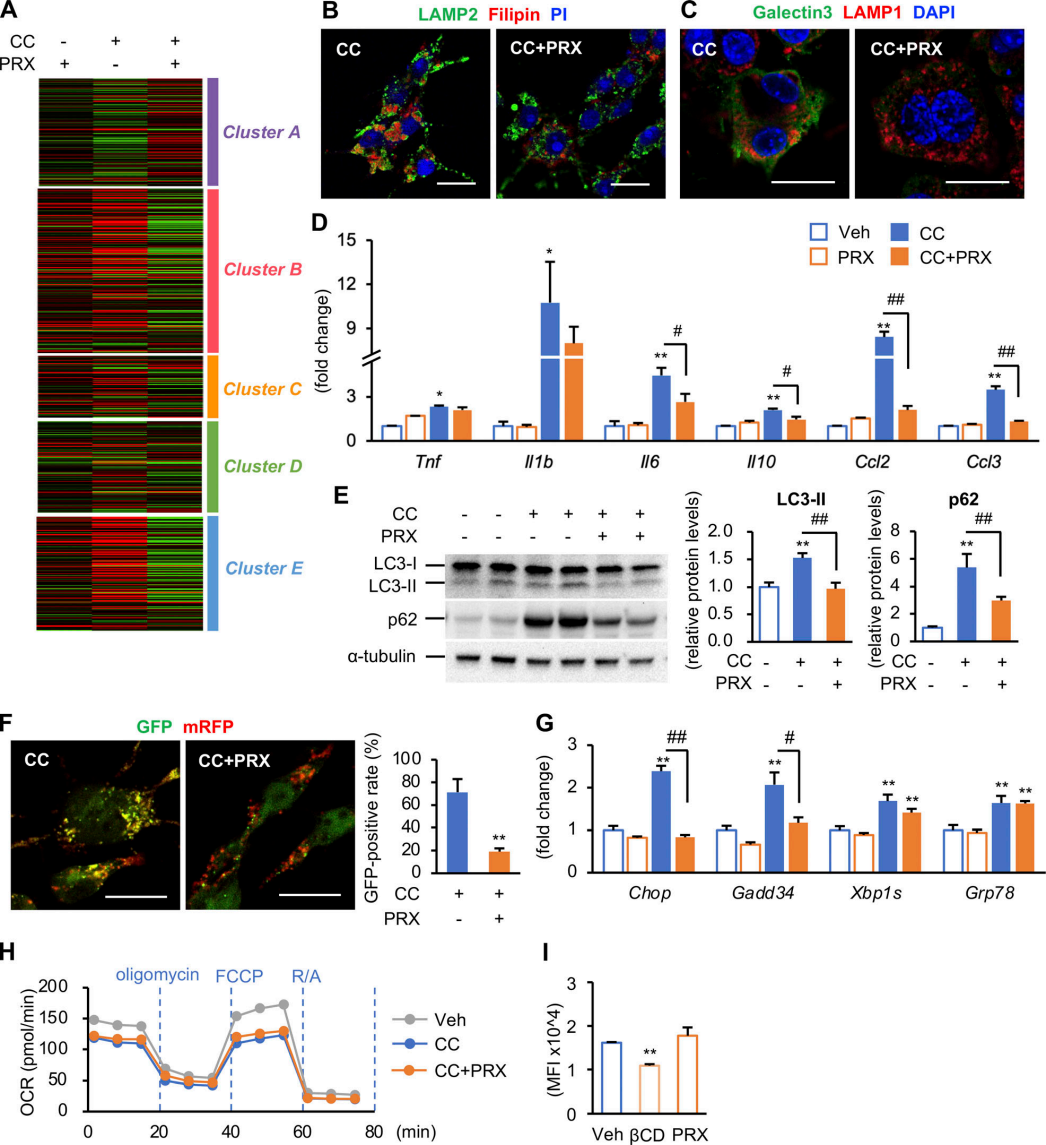
**Effect of cholesterol crystals (CCs) on lysosomal stress functions in RAW264 macrophages. (A)** RAW264 macrophages treated with CC and βCD-PRX for 24 h were subjected to RNA-seq analysis (*n* = 4). Heatmap showing fold changes of the genes extracted by an unsupervised hierarchical clustering of hepatic macrophages shown in Fig. 6 G compared with vehicle-treated RAW264 macrophages. **(B and C)** Representative images of immunostaining of RAW264 macrophages treated with CC and βCD-PRX. **(B)** LAMP2 (a lysosome marker, green), filipin (free cholesterol, red), and PI (nuclei, blue). **(C)** Galectin 3 (green), LAMP1 (red), and DAPI (blue). Scale bars, 20 μm. **(D)** mRNA expression levels of genes related to inflammatory cytokines and chemokines. *n* = 4. *P < 0.05, **P < 0.01 versus Veh; ^#^P < 0.05, ^##^P < 0.01. **(E)** Western blot analysis of LC-3 and p62 protein levels. *n* = 4. **P < 0.01 versus Veh; ^##^P < 0.01. **(F)** Images of RAW264 macrophages transiently expressing mRFP-GFP-LC3 treated with CC and βCD-PRX for 24 h and GFP-positive ratio of mRFP-positive puncta. **P < 0.01 versus CC. **(G)** mRNA expression levels of genes related to endoplasmic reticulum stress. *n* = 4. **P <0.01 versus Veh; ^#^P < 0.05, ^##^P < 0.01. **(H)** Oxygen consumption rate (OCR) of RAW264 macrophages treated with CC and βCD-PRX for 18 h. *n* = 2. FCCP, carbonyl cyanide 4-(trifluoromethoxy)phenylhydrazone; R/A, rotenone/antimycin A. **(I)** Effect of HP-βCD (βCD) and βCD-PRX on the levels of lipid raft integrity evaluated by FACS analysis using cholera toxin subunit B, a marker for lipid rafts. *n* = 3–4. **P < 0.01 versus Veh. Data and images are representative of two (B, C, F, H, and I) or three (D, E, and G) independent experiments. Error bars represent means ± SEM. Source data are available for this figure: SourceData FS4.

Next, we examined the molecular mechanisms of action of βCD-PRX using RAW264 macrophages because RAW264 macrophages showed similar gene expression profiles (RNA-seq data, *n* = 4) and immunostaining patterns to hepatic macrophages from steatotic livers when treated with cholesterol crystals (Fig. 8, A and B; and Fig. S4, A–D). Acridine orange emits fluorescence in acidic environments, indicating the intact function of lysosome membranes (Kanamori et al., 2021). Flowcytometric analysis revealed increased side scatter intensity, reflecting the uptake of cholesterol crystals (Ioannou et al., 2019), and decreased fluorescence intensity of acridine orange, indicating lysosomal dysfunction (Fig. 8 C). βCD-PRX treatment prevented cholesterol overload–induced lysosomal dysfunction (Fig. 8 C) and restored lysosomal function even after cholesterol crystals were once taken up by macrophages (Fig. 8 D). βCD-PRX treatment also suppressed the otherwise increased protein levels of microtubule-associated protein 1 light chain 3-II form (LC3-II), which localizes autophagosomal membrane, and p62, a selective substrate for autophagic protein degradation (Fig. S4 E). Similar results were obtained using a tandem fluorescence-tagged vector (mRFP-GFP-LC3), which emits both mRFP and GFP signals in autophagosomes and only an mRFP signal in autolysosomes (Kimura et al., 2007; Fig. S4 F). All these data indicate that βCD-PRX treatment protects against cholesterol crystal–induced lysosomal stress/injury in macrophages. Although cholesterol loading affected endoplasmic reticulum stress and mitochondrial function, βCD-PRX treatment showed only limited effects (Fig. S4, G and H).

**Figure S5.**
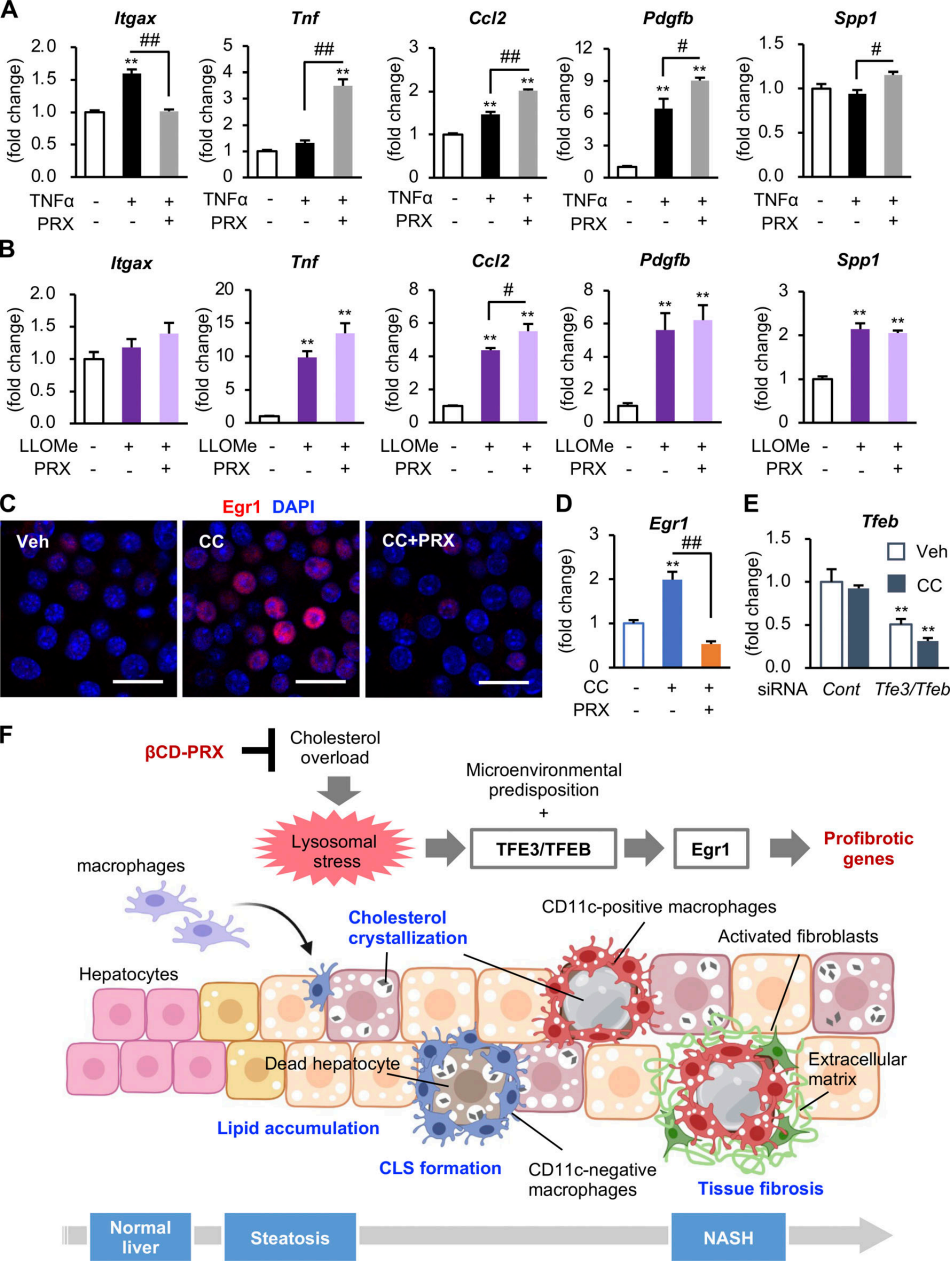
**Molecular mechanism of action of βCD-PRX. (A and B)** Effect of βCD-PRX on TNFα (20 μg/ml, 24 h; A) and L-leucyl-L-leucine methyl ester (LLOMe, 1 mM, 3 h), an artificial lysosomal membrane damage reagent (B). *n* = 4. **P < 0.01 versus Veh; ^#^P < 0.05, ^##^P < 0.01. **(C and D)** Representative images of Egr1 immunostaining (C) and mRNA expression of Egr1 (D) in RAW264 macrophages treated with cholesterol crystals (CCs) and βCD-PRX for 24 h *n* = 4. **P < 0.01 versus Veh; ^##^P < 0.01. **(E)** mRNA expression in RAW264 macrophages treated with CC for 6 h under knockdown of Tfe3 and Tfeb. *n* = 4. **P < 0.01 versus Cont-Veh. Data and images are representative of three (A) or two (B–E) independent experiments. Error bars represent means ± SEM. **(F)** Potential role of free cholesterol overload in profibrotic transformation of hepatic macrophages interacting with dead hepatocytes. During the course of NASH development, macrophages aggregate around dead hepatocytes with CCs in the lipid droplets, and macrophages engulf the corpses of hepatocytes and remnant lipids. In the microenvironment of steatotic livers, macrophages undergo profibrotic activation at least partly through TFE3/TFEB-Egr1 axis. βCD-PRX excretes free cholesterol from lysosomes and reverses the phenotypic changes of macrophages. Created with BioRender.

We next compared the effect of βCD-PRX with that of HP-βCD unthreaded in linear polymer and simvastatin, a 3-hydroxy-3-methylglutaryl-CoA reductase inhibitor. Treatment with HP-βCD and simvastatin showed only limited or no effects on intracellular cholesterol accumulation and cholesterol excretion in the media (Fig. 8, E and F). In line with this result, the expression of TFE3-target genes was not suppressed by HP-βCD or simvastatin (Fig. 8 G). FACS analysis using cholera toxin subunit B, a lipid raft marker, revealed that HP-βCD disrupted the integrity of lipid rafts, but βCD-PRX did not (Fig. S4 I). Moreover, we examined the effect of βCD-PRX on cholesterol-independent inflammatory stimuli, such as TNFα and L-leucyl-leucine methyl ester, a lysosome-destabilizing agent, and found that βCD-PRX treatment did not suppress mRNA expression of proinflammatory and profibrotic genes under these stimuli (Fig. S5, A and B). These results suggest that βCD-PRX exerts its anti-profibrotic effects via enhanced lysosomal cholesterol excretion and subsequent amelioration of lysosomal stress/injury.

Consistent with the data using hepatic macrophages isolated from steatotic macrophages (Figs. 6 and 7), increased mRNA expression and nuclear translocation of Egr1 were observed in RAW264 macrophages treated with cholesterol crystals (Fig. S5, C and D). Knockdown of Egr1 almost completely inhibited the expression of osteopontin and PDGF without affecting the expression of lysosome-related genes (Fig. 8 H). We also found that knockdown of MiT/TFE transcription factors (TFE3/TFEB [transcription factor EB]) markedly suppressed the expression of *Egr1*, lysosome-related genes, and Egr1-target genes including *Spp1* and *Pdgfb* (Fig. 8 I and Fig. S5 E). These observations suggest that TFE3/TFEB drives fibrogenic pathways at least partly through activation of Egr1.

### Phagocytosis of dead cells induces lysosomal dysfunction in macrophages

**Figure 9.**
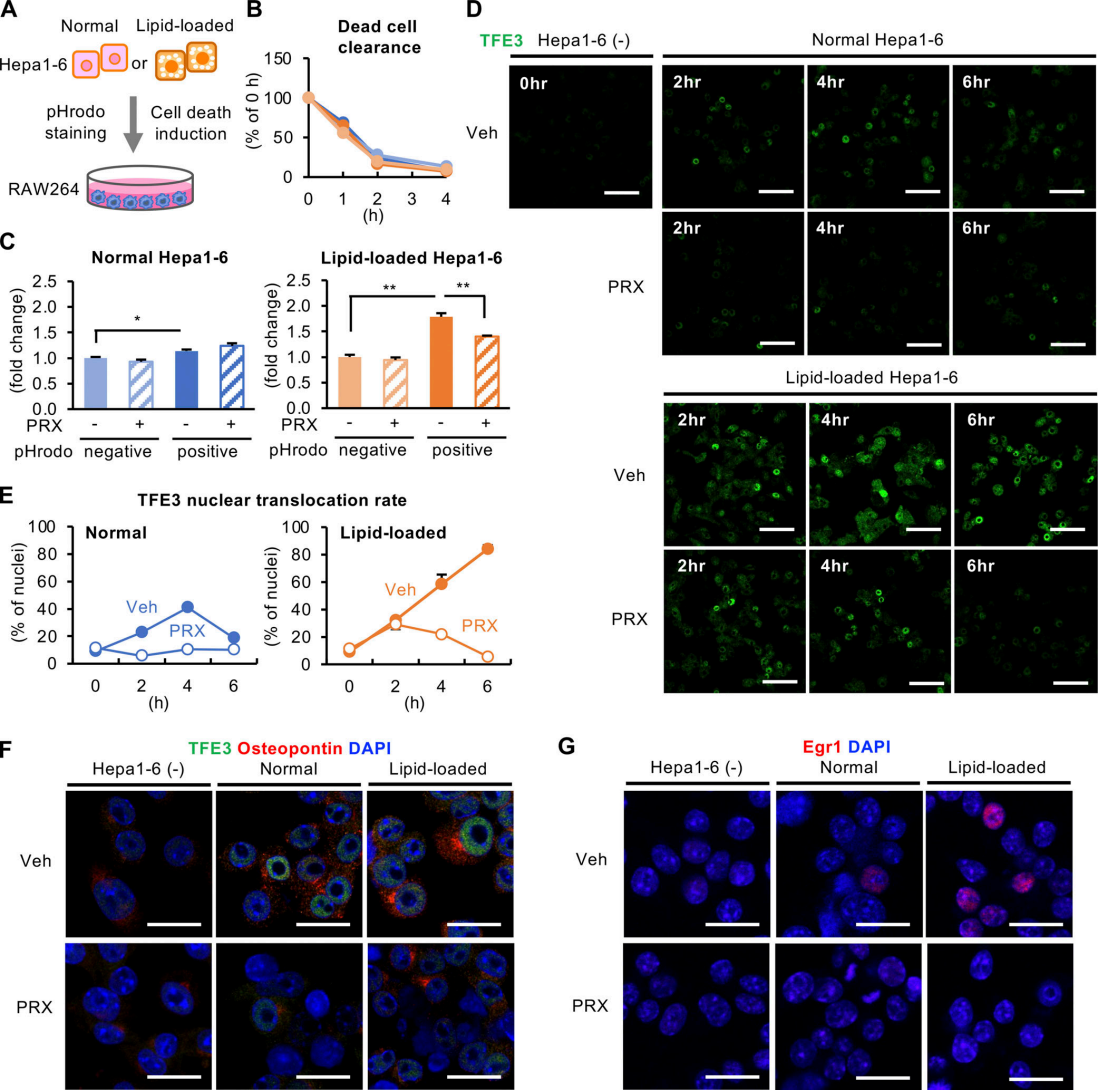
**Phagocytosis of dead cells induces lysosomal dysfunction and profibrotic changes in macrophages. (A)** Experimental protocol for macrophage phagocytosis of dead cells. Hepa1-6 cells were incubated with the inclusion complex of cholesterol with randomly methylated βCD (50 μM) and oleic acid (500 μM) for 48 h. Unstimulated (indicated as normal) and lipid-loaded Hepa1-6 cells were stained with pHrodo, which emits fluorescence in an acidic environment in lysosomes, and induced necrotic cell death by freeze and thaw. RAW264 macrophages were incubated with dead Hepa1-6 cells for the indicated time. **(B)** Clearance rate was evaluated by the number of dead cells in the media at the indicated time. Orange, normal Veh; pale orange, normal PRX; blue, lipid-loaded Veh; pale blue, lipid-loaded PRX. *n* = 4. **(C)** Macrophages were incubated with dead cells with or without βCD-PRX for 6 h and pHrodo-positive and -negative macrophages were subjected to measurement of free cholesterol content. *n* = 3–4. *P < 0.05, **P < 0.01. **(D)** Immunostaining of TFE3 (green) in RAW264 macrophages after supplementation of dead cells and βCD-PRX. hr, hour. **(E)** Time course of TFE3 nuclear translocation. **(F and G)** Immunostaining of TFE3 (green), Osteopontin (red; F), and Egr1 (red; G) in RAW264 macrophages. Scale bars, 20 μm. Data and images are representative of two independent experiments. Error bars represent means ± SEM.

Next, we examined the involvement of phagocytosis of dead cells in lysosomal dysfunction in macrophages. Unstimulated (indicated as normal) or lipid-loaded Hepa1-6 cells were stained with pHrodo, rendered necrotic by freeze and thaw, and then added to RAW264 macrophages (Fig. 9 A). There was no difference in the clearance of dead cells, regardless of the type of dead cells or the existence of βCD-PRX (Fig. 9 B). The free cholesterol content was markedly increased in macrophages by adding lipid-loaded Hepa1-6 compared to normal Hepa1-6, and the increase was considerably suppressed by βCD-PRX (Fig. 9 C). After engulfment of lipid-loaded Hepa1-6 cells, nuclear translocation of TFE3 was observed in a time-dependent manner, whereas it was observed only transiently and mildly after engulfment of normal Hepa1-6 cells (Fig. 9, D and E). In parallel with these changes, nuclear translocation Egr1, along with increased expression of osteopontin, was observed in macrophages phagocytosing lipid-loaded Hepa1-6 cells (Fig. 9, F and G). Taken together, these findings suggest that phagocytosis of dead cells, as well as cholesterol crystals, induces lysosomal stress in macrophages to acquire profibrotic properties.

## Discussion

Evidence has accumulated regarding the macrophage subsets responsible for the development of liver fibrosis termed SAM, NASH-associated macrophage, and lipid-associated macrophage (Ramachandran et al., 2019; Remmerie et al., 2020; Xiong et al., 2019). Recently, Fabre et al. reported that CD9 and triggering receptor expressed on myeloid cells 2–positive macrophages expressing Spp1, glycoprotein nonmetastatic melanoma protein B, fatty acid binding protein 5, and CD63 are highly profibrotic and enriched in the fibrotic niche across species and organs using multiple single-cell RNA-seq datasets (Fabre et al., 2023). We have also found a macrophage subset localizing to CLS in murine NASH models, harboring gene expression patterns similar to the above profibrotic macrophages, based on a completely different approach (Itoh et al., 2017). In this study, using a unique supramolecule, βCD-PRX, we demonstrated that lysosomal-free cholesterol accumulation triggers lysosomal stress/injury and profibrotic activation of macrophages. Of note, such lysosomal stress was also observed in the macrophages constituting CLS in human NASH (Fig. 1; Kanamori et al., 2021). Several studies have pointed to the foamy appearance of hepatic macrophages in hyperlipidemic mice fed a WD, in which cholesterol overload in macrophages has been considered as a result of high levels of circulating low-density lipoprotein similar to atherosclerotic plaques (Sano et al., 2004; Yoshimatsu et al., 2004). MC4R-KO mice exhibited hypercholesterolemia, whereas cholesterol accumulation and lysosomal stress were observed only in the CD11c-positive macrophages in CLS, suggesting that free cholesterol is derived from dead hepatocytes. Accordingly, this study sheds light on the novel mechanism for lipotoxicity as the pathogenesis of NASH and provides evidence that the microenvironmental milieu in the liver modulates stress response of macrophages, thereby activating profibrotic programs during the development of NASH.

As HP-βCD promotes cholesterol removal from the cells, the efficacy of HP-βCD has been reported in mouse models of Niemann-Pick type C (NPC) disease and atherosclerosis (Liu et al., 2009; Ory et al., 2017; Zimmer et al., 2016). However, a substantial amount of HP-βCD is required ranging from 4,000 to 8,000 mg/kg body weight to achieve therapeutic effects. In addition, a high prevalence of adverse effects such as acute toxicity, pulmonary injury, and ototoxicity may hinder the clinical application of HP-βCD when it is used for lifestyle-related diseases that require high safety in the long term. In contrast, βCD-PRX is designed to avoid such toxic effects because βCD-PRX does not directly act on free cholesterol of the plasma membranes (Tamura and Yui, 2014). In this study, we confirmed that HEE-modified βCD-PRX is hepatotropic, low toxic, and capable of expelling free cholesterol from macrophages at a low dose (30 mg/kg) in vivo. Although βCD-PRX was distributed to hepatocytes as well as macrophages, it did not affect the cholesterol content in hepatocytes. This is probably because cholesterol is mainly stored in lipid droplets in hepatocytes, whereas βCD-PRX is delivered to lysosomes (Tamura et al., 2016). Cholesterol-lowering medications, such as statins and ezetimibe, have been tested in NAFLD/NASH patients because of the high prevalence of hypercholesterolemia and cardiovascular diseases (Horn et al., 2022). Statins inhibit 3-hydroxy-3-methylglutaryl-CoA reductase, the rate-limiting enzyme of cholesterol synthesis in hepatocytes, and ezetimibe inhibits intestinal cholesterol absorption by binding to the NPC-like 1 sterol transporter, both of which are expected to reduce the cholesterol burden on the liver. However, large randomized placebo-controlled trials are still lacking to prove their efficacy in liver fibrosis. On the other hand, βCD-PRX targets accumulated free cholesterol in lysosomes, so that lysosomal function is restored in macrophages constituting CLS, thereby leading to suppression of profibrotic activation. Thus, βCD-PRX could be a novel therapeutic agent for NASH with a unique mechanism of action.

During the development of NASH, KCs derived from yolk sac are activated and partially undergo cell death, while monocyte-derived macrophages, in turn, occupy the niche and differentiate into those with KC-like signatures (F4/80^hi^ CD11b^lo^; Seidman et al., 2020; Xiong et al., 2019). In this study, since the F4/80^hi^ CD11b^lo^ macrophage fraction was separated regardless of their origin, they may include both yolk sac–derived KCs and monocyte-derived macrophages. Given that βCD-PRX affected the KC-like signature, improvement of lysosomal function would regulate cell death or macrophage differentiation. Of note, profibrotic genes were upregulated only in cultured macrophages isolated from steatotic livers in response to lysosomal stress, but not from normal livers, suggesting that the predisposition of macrophages in the microenvironment of steatotic livers is required for sensing metabolic stress and subsequent activation of profibrotic pathways. In line with this, Seidman et al. reported that hepatic macrophages possess disease-specific enhancer landscapes that suppress KC identity and promote SAM-like phenotype (Seidman et al., 2020). Intriguingly, we demonstrated a novel mechanism that cholesterol overload induces activation of TFE3/TFEB, leading to upregulation of Egr1 and subsequent activation of profibrotic pathways. Since TFE3/TFEB plays important roles in various cell types, future research would clarify the significance of TFE3/TFEB and/or Egr1 in macrophages in the pathogenesis of NASH. On the other hand, as shown in Fig. 5 B, treatment with βCD-PRX significantly but partially suppressed the number of TFE3-positive macrophages in CLS, suggesting the involvement of other factors in the induction of lysosomal stress in macrophages during the development of NASH. For instance, changes in lipid composition, such as cholesterol or sphingosine levels and reactive oxygen species, can lead to increased lysosomal membrane permeabilization (Gómez-Sintes et al., 2016; Kirkegaard et al., 2010). Our previous data revealed that iron accumulation induces lysosomal stress and profibrotic activation in CLS-forming macrophages (Kanamori et al., 2021). It is likely that multiple metabolic changes, including cholesterol and iron, are involved in lysosomal stress/injury observed in this process.

A substantial amount of cholesterol is accumulated in macrophages after ingestion of apoptotic cells, and lysosomal acid lipase is required to hydrolyze esterified cholesterol from ingested apoptotic cells to activate liver X receptor (Mota et al., 2021; Viaud et al., 2018). In this study, we observed increased content of free cholesterol in RAW264 macrophages after engulfment of Hepa1-6 cells possessing lipid droplets filled with esterified cholesterol, lysosomal degradation of esterified cholesterol results in free cholesterol overload to macrophages, which induced Egr1 activation. Our in vitro and in vivo data support the notion that accumulated cholesterol in macrophages is derived from dead hepatocytes and activates profibrotic pathways upon phagocytosis, which could be reversed by βCD-PRX treatment. However, the limitation of this study is that phagocytosis assay was conducted with immortalized cell lines, and the experimental settings are not sufficient to recapitulate the cellular interaction in CLS. For the next step, it is important to investigate the molecular mechanisms of how dead cell clearance induces profibrotic changes in macrophages, and how free cholesterol crystalizes in lipid droplets of dying hepatocytes.

In summary, we demonstrated that cholesterol derived from dead hepatocytes induces lysosomal dysfunction in the macrophages constituting CLS, leading to upregulation of profibrotic genes, thereby promoting liver fibrosis (Fig. S5 F). Treatment with βCD-PRX ameliorated liver fibrosis at least partly through decreasing free cholesterol content in macrophages and suppressing the activation of profibrotic pathways in a NASH model. This study provides evidence that lysosomal cholesterol overload triggers macrophage phenotypic changes and promotes the development of NASH, which could be a novel therapeutic target for NASH.

## Materials and methods

### Reagents

All reagents were purchased from Sigma-Aldrich or Nacalai Tesque unless otherwise noted. Chemically modified βCD-PRXs were synthesized and characterized as described previously (Nishida et al., 2016; Ohashi et al., 2021; Tamura and Yui, 2014, 2015; Tonegawa et al., 2020; Zhang et al., 2021). The number of threading βCD, the number of modified functional groups, and the number-average molecular weight of each chemically modified βCD-PRX are shown in Table S2. The HEE-group modified βCD-PRX used in the mouse experiment of NASH was as follows: the number of threading βCDs, 11.8; the number of modified HEE groups, 5.29 per threaded βCD (62.4 per βCD-PRX); and the average molecular weight per βCD-PRX, 27,900.

### Animals

MC4R-KO mice on a C57BL/6J background were kindly provided by Joel K. Elmquist (University of Texas Southwestern Medical Center, Dallas, TX, USA). Age-matched C57BL/6J WT mice were purchased from CLEA Japan. 8-wk-old male MC4R-KO and WT mice were fed a WD (D12079B; 468 kcal/100 g, 41% energy as fat, 34.0% sucrose, and 0.21% cholesterol; Research Diets) for 24 wk to induce NASH. WT mice were maintained on a standard diet (CE-2; CLEA) for the same period. HC diet–induced NASH model, 8-wk-old male WT mice were fed an HC diet (D09100310; 40 kcal% fat, 20 kcal% fructose, 2% cholesterol; Research Diets) for 20 wk. At the end of the experiment, the animals were sacrificed under anesthesia when fed ad libitum.

### βCD-PRX administration

βCD-PRX was subcutaneously administered using osmotic minipumps at a dose of 30 mg/kg/d (Alzet model 2002; Palo Alto). After 18-wk WD or HC feeding, osmotic minipumps filled with βCD-PRX or normal saline were implanted under the back skin of mice. They were kept on the WD or HC for an additional 6 wk, with the replacement of minipumps every 2 wk. To analyze the cellular distribution of βCD-PRX in the liver, βCD-PRX was administered intraperitoneally at the indicated doses, and the livers were analyzed after 24 h. The Cy5.5-labeled chemically modified βCD-PRXs were administered subcutaneously at 200 mg/kg, and the fluorescence intensities of each tissue were analyzed after 24 h.

### Blood analysis

Blood glucose concentrations were measured using a blood glucose test meter (Glutest PRO R; Sanwa Kagaku). Serum concentrations of ALT, tryglyceride (TG), and TC were measured by the biochemical analyzer (DRI-CHEM NX500V; Fujifilm). Serum concentrations of insulin and cytokines were determined using the ELISA kit (Morinaga Co. Ltd.). Serum concentrations of TNFα, IL-1β, MCP-1, Osteopontin, and PDGF-BB were measured by each ELISA kit (R&D).

### FACS analysis and sorting experiments

The mice were perfused with PBS or HBSS without calcium and magnesium to remove blood from the liver. To isolate hepatocytes, the livers were dispersed in HBSS with calcium and magnesium supplemented with 1 mg/ml type IV collagenase (Sigma-Aldrich), and the cell suspensions were centrifuged at 50–100 × *g* for 2 min. The livers were digested using a gentleMACS dissociator (Miltenyi Biotech) to isolate the non-parenchymal cell fraction, and cell debris was removed by Percoll density gradient centrifugations. Non-parenchymal cells were stained with antibodies against CD45 (30-F11), F4/80 (BM8), CD11b (M1/70), CD3 (17A2), CD11c (N418), CD146 (ME-9F1), Ly6G (1A8), and SiglecF (S17007A; BioLegend). Dead cells were separated using 7-AAD or DAPI. Cells were analyzed using FACSCanto II (BD Biosciences) or sorted using FACSAria II (BD Biosciences). Cell plots were analyzed using FlowJo v10 software.

### Measurement of lipid content of livers and isolated cells

Total lipids in the liver were extracted using chloroform/methanol. The total cholesterol and triglyceride concentrations in the livers were measured by enzymatic assay kits (Fujifilm Wako Pure Chemicals). The cellular cholesterol content of isolated primary macrophages, hepatocytes, and RAW264 was measured by gas chromatography–mass spectrometry (GC-MS). Briefly, the cells were suspended in PBS (500 μl), and 5α-cholestane (50 μg/ml in pyridine, 12 μl) was added as an internal standard. Cholesterol was extracted with chloroform and methanol, and the organic phases were collected and evaporated to dryness via nitrogen flow. The dried samples were dissolved in dehydrated pyridine (150 μl; Fujifilm Wako Pure Chemical) and derivatized with *N*-methyl-*N*-(trimethylsilyl)trifluoroacetamide (50 μl) for 30 min at 60°C for the quantification of cholesterol. GC-MS measurements were performed on a GCMS-QP2020 (Shimadzu) equipped with an AOC-20i autoinjector and a DB-5MS capillary column (30 m × 0.25 mm internal diameter, 0.25 μm phase thickness; Agilent Technologies). Ultrahigh-purity helium (>99.999%) was used as the carrier gas. Sample solutions (1 μl) were injected in splitless mode, and the measurements were performed in a selected-ion monitoring mode. The oven temperature was held initially at 50°C for 2 min (0–2 min), increased to 260°C at a rate of 40°C/min (2–7.25 min), increased further to 310°C at a rate of 2.5°C/min (7.25–27.25 min), and finally held at 310°C for 2 min (27.25–29.25 min). The ions used for the quantification were as follows: cholesterol (22.010 min) *m/z* = 458 and 5α-cholestane (17.405 min) *m/z* = 217. For the quantification of cholesteryl esters, the samples were hydrolyzed with KOH (150 mg/ml in ethanol) for 1 h at 70°C to quantify total cholesterol. The amount of cholesteryl esters was determined by subtracting the free cholesterol values from the total cholesterol values. The amounts of cellular free cholesterol and esterified cholesterol were expressed by normalizing with the cell number.

### Histological analysis

The livers were fixed with 10% neutral-buffered formalin and embedded in paraffin. 4-μm-thick sections of the liver were stained with Sirius red. Immunohistochemical staining was performed for F4/80 (MCA497GA; Serotec), desmin (ab15200; Abcam), CTSD (ab75852; Abcam), osteopontin (AF808; R&D), and type III collagen (1330-01; SouthernBiotech). Positive areas for Sirius red, osteopontin, desmin, and type III collagen were measured on the whole area of each slice using ImageJ software (National Institutes of Health). For immunofluorescent staining, the livers were embedded in an OCT compound and frozen in liquid nitrogen. 10-μm-thick frozen sections were stained with antibodies against F4/80, TFE3 (HPA023881; Sigma-Aldrich), CD11c (14-0114; eBioscience), Clec4f (MAB2784; R&D), Tim4 (130002; BioLegend), and secondary antibodies. Sections were mounted in Vectashield mounting medium with DAPI (Vector Labs) and photographed under identical settings for each staining using a C2 confocal microscope (Nikon). TFE3-positive ratio was quantified by counting the number of TFE3-positive nuclei out of DAPI spots in all the CLS observed on each slice. CD11c-positive CLS was determined when some or all of the macrophages forming CLS expressed CD11c. To observe cholesterol crystals in NASH livers from bone marrow chimeric MC4R-KO mice with GFP-Tg mice fed a WD for 20 wk, 1-mm-thick sliced liver samples were fixed with 4% paraformaldehyde, embedded in OCT compound, and frozen in liquid nitrogen. 10-μm-thick frozen sections were dried and observed using BX53 microscope with a polarizing filter (Olympus).

### Electron microscopy analysis

MC4R-KO mice fed a WD for 20 wk were perfused with 2.5% glutaraldehyde and 4% paraformaldehyde in 0.1 M phosphate buffer, pH 7.4. Liver tissue was cut into 1-mm cubes and postfixed in 2.5% glutaraldehyde and 4% paraformaldehyde overnight. Fixed samples were cut with a microslicer at 500 μm thick. The slices were then fixed with 1% OsO4 for 1 h, dehydrated in graded ethanol, and flat-embedded on siliconized glass slides in epoxy resin. Serial ultrathin sections of the livers at 70-nm thickness were mounted on pieces of silicon wafers and contrasted with uranyl acetate and lead citrate. Scanning electron microscopy images were obtained using a backscattered electron detector (BED-C; voltage: 7 kV; PC current, 1.8 nA; work distance: 6) in a JSM-7900F scanning electron microscope (JEOL).

### Cholesterol preparation

Cholesterol crystals were generated as previously described (Emanuel et al., 2014). Briefly, cholesterol was dissolved in ethanol at a concentration of 50 mg/ml by heating to 60°C. Cholesterol crystals were formed at −30°C and centrifuged at 10,000 × *g* for 20 min. The crystals were resuspended in PBS at 50 mg/ml and sonicated extensively. The inclusion complex of cholesterol with randomly methylated βCD (βCD/Chol) was prepared by stirring cholesterol powder (38.6 mg) in 100 mM randomly methylated βCD solution for 24 h at room temperature. The solution was passed through a 0.22-μm filter before use.

### Solubility of cholesterol by inclusion complexation with βCDs

The cholesterol solubility of differentially modified βCD-PRXs was measured using high-performance liquid chromatography, according to the previous reports (Tamura et al., 2016).

### Cell culture

RAW264 murine macrophage cells and Hepa1-6 murine hepatoma cells were maintained in Dulbecco’s minimal essential media supplemented with 10% FBS and antibiotics. For primary hepatic macrophage culture, non-parenchymal cell fractions isolated from the livers of WT mice fed an SD and MC4R-KO mice fed a WD for 6–10 wk were stained with PE-conjugated F4/80 antibody (REA126; Miltenyi) and MojoSort Mouse anti-PE Nanobeads (BioLegend), and separated using an autoMACS separator (Miltenyi; Kanamori et al., 2021). Primary hepatocytes were isolated by centrifugation at 50–100 × *g* from the liver suspensions of WT mice fed an SD and MC4R-KO mice fed a WD for 10 d. Primary hepatic macrophages and hepatocytes were seeded on collagen-coated plates. The cytotoxicity of chemically modified βCD-PRXs on RAW264 macrophages was assessed using Cell TiterGlo 2.0 (Promega). RAW264 macrophages and hepatic macrophages were treated with cholesterol crystals (500 μg/ml) for the indicated times with or without βCD-PRX (1 mM), HP-βCD (1 mM), or simvastatin (1 μM). Hepa1-6 cells were incubated with βCD/Chol (50 μM) and oleic acid (500 μM) for 48 h to produce lipid droplets in the cytoplasm.

### Cell transfection experiments

RAW264 macrophages were reverse-transfected with small interfering RNA (siRNA) targeting TFE3 (Invitrogen), TFEB (Sigma-Aldrich) for 48 h, and Egr1 (Invitrogen) for 24 h using lipofectamine RNAiMax (Invitrogen). After siRNA transfection, cells were treated with cholesterol crystals with or without βCD-PRX for 6 h. RAW264 macrophages plated on glass bottom dishes were transfected with plasmid DNA encoding mRFP-GFP tandem fluorescence-tagged LC3 (21074; Addgene) using lipofectamine 3000 (Invitrogen). After 48 h incubation, the cells were treated with cholesterol crystals for 24 h and analyzed by C2 confocal microscope.

### Immunocytochemistry of macrophages

Primary hepatic macrophages and RAW264 macrophages were seeded on chambered cover glasses (Nalge Nunc) and stimulated as indicated. Cells were fixed with 4% paraformaldehyde and stained with anti-LAMP1 (121601; BioLegend), LAMP2 (ab13524; Abcam), Galectin-3 (14979-1-AP; Proteintech), TFE3 (HPA023881; Sigma-Aldrich), Egr1 (4153; Cell Signaling), osteopontin (AF808; R&D), and Filipin (50 μg/ml; Polysciences). Photographs were captured under identical settings for each staining. TFE3-positive ratio was quantified by counting the number of TFE3-positive nuclei out of DAPI spots.

### Evaluation of lysosomal function using acridine orange

RAW264 macrophages were treated with cholesterol crystals (500 μg/ml) and βCD-PRX for indicated times. Acridine orange was added at a concentration of 5 μg/ml for 30 min before FACS analysis.

### RNA extraction and quantitative real-time PCR

Total RNA was extracted from the liver or cultured cells using Sepasol reagent or PureLink RNA Micro Kit (Invitrogen). Quantitative real-time PCR was performed with the StepOnePlus Real-time PCR System using the Fast SYBR Green Master Mix Reagent (Applied Biosystems) as previously described. The primers used in this study are listed in Table S6. Data were normalized to 36B4 levels and analyzed using the comparative Ct method.

### RNA-seq analysis

Hepatic macrophages isolated from normal and steatotic livers were treated with cholesterol crystals for 24 h (*n* = 2), and RAW264 macrophages were treated with cholesterol crystals with or without βCD-PRX (*n* = 4). Total RNA was purified using RNeasy MiniElute Cleanup Kit (Qiagen). RNA was quantified with Qubit RNA BR Assay Kit (Invitrogen) and the integrity was evaluated with Agilent Bioanalyzer 2100 (Agilent Technologies). RNA-seq libraries were constructed by NEBNext Poly (A) mRNA Magnetic Isolation Module (NEB) and MGIEasy RNA Directional Library Prep Set (MGI), according to the manufacturer’s instructions. Libraries were sequenced with 150 bp paired-end reads on a DNBSEQ-G400 sequencer (MGI Tech). Low-quality reads and adapter sequences were trimmed using fastp software (Chen et al., 2018). The reads were mapped to the mm10 reference genome using HISAT2 (Kim et al., 2019) followed by transcript assembly and quantification using StringTie (Pertea et al., 2015). Genes expressed below 0.5 counts per million were filtered out from analysis. Fold changes of gene expression levels were analyzed between hepatic macrophages from normal and steatotic livers or RAW264 macrophages treated with cholesterol crystals with or without βCD-PRX using iDEP v.0.94 (Ge et al., 2018), Metascape, and IPA (QIAGEN).

### Western blotting analysis

RAW264 macrophages were lysed in radioimmunoprecipitation assay buffer (FujiFilm Wako) supplemented with Protease Inhibitor Cocktail (Sigma-Aldrich). Proteins were separated by SDS-PAGE and immunoblotted with anti-LC3 antibody (PM0369; MBL), anti-p62 antibody (PM045; MBL), and anti-α-tubulin antibody (T5168; Sigma-Aldrich). Immunoblots were detected and analyzed with ECL Prime (Cytiva) and ChemiDoc XRS Plus (Bio-Rad).

### Human study

Japanese NAFLD/NASH patients who had sustained liver dysfunction, dyslipidemia, and insulin resistance were recruited at Yamaguchi University Hospital and Yokohama City University Hospital. We measured body mass index and serum parameters according to the standard procedures. LSM and CAP values were determined by FibroScan (Echosens). Liver samples were obtained by ultrasound-guided liver biopsy to evaluate liver histology. NAS score and fibrosis stage were assessed according to the NASH clinical research network scoring system. Formalin-fixed and paraffin-embedded liver specimens were stained with antibodies against CD68 (M0876; Dako), CD11c (EP1347Y, Abcam), and CTSD.

### Statistics

Data are presented as mean ± standard error of mean (SEM), and statistical significance was set at P < 0.05. Statistical analysis was performed using analysis of variance, followed by Tukey-Kramer test. Two-tailed unpaired Student’s *t* test was used to compare the two groups. Statistical analyses were performed with JMP Pro 15 (SAS Institute, Cary, NC, USA). In human study, box plots were created for CAP values by NAS and fibrosis stage, and LSM values by fibrosis stage to evaluate associations. Correlations were evaluated by calculating Spearman’s rank correlation coefficient. To evaluate the association of NAS, fibrosis stage, CAP, and LSM with respect to CLS number, we used a generalized linear model with CLS number as the dependent variable and the logarithm of the link function. In this analysis, a first-order linear model was constructed for NAS, fibrosis stage, CAP, and LSM, and a model including a quadratic term to evaluate the curvilinear relationship. R2 values were calculated as the goodness of fit of the models.

### Study approval

All animal experiments were approved by the Guidelines for the Care and Use of Laboratory Animals of Nagoya University and the Animal Care and Use Committee, Research Institute of Environmental Medicine, Nagoya University (No. 20253), and the Institutional Animal Care and Use Committee of Tokyo Medical and Dental University (A2018-158A, A2020-114C and A2022-072A). All animal experiments were carried out according to the Animal Research: Reporting of In Vivo Experiments guidelines. The clinical study complied with the principles of the Declaration of Helsinki. The protocol was approved by the Ethical Committee for Human and Genome Research of Research Institute of Environmental Medicine, Nagoya University (No. 388), the Medical Research Ethics Committee of Tokyo Medical and Dental University (M2015-544), the Institutional Review Board of Yamaguchi University Hospital (2020-063), and the Ethics Committee of Yokohama City University Hospital (B151101010). Although written informed consent was not obtained for the current study, we obtained approval from the Ethics Committee/Institutional Review Board of each institution based on the Japanese Ethical Guidelines for Clinical Studies, disclosed detailed information on the study protocol, and provided all participants with an opportunity to refuse their inclusion in the study.

### Online supplemental material

Fig. S1 shows the relationship between crown-like structure number and clinical parameters in NAFLD/NASH subjects (related to Table S1). Fig. S2 shows the effect of βCD-PRX on cholesterol accumulation in hepatocytes. Fig. S3 shows the effect of βCD-PRX on liver fibrosis in a mouse model of NASH with high-fat and HC diet. Fig. S4 shows the effect of cholesterol crystal–indued lysosomal stress and the impact on cellular function (related to Fig. 8). Fig. S5 shows the molecular mechanism of action of βCD-PRX. Table S1 shows clinical data of NAFLD/NASH patients. Table S2 shows molecular characteristics of chemically modified βCD-PRXs (related to Fig. 2). Table S3 shows serological parameters of WT mice treated with chemically modified βCD-PRXs (related to Fig. 2). Table S4 shows serum cytokine levels in MC4R-KO mice treated with βCD-PRX for 6 wk (related to Fig. 4). Table S5 shows the effect of 6-wk βCD-PRX treatment on serological parameters of WT mice fed an HC diet for 20 wk (related to Fig. S3). Table S6 lists primers used in this study.

## Supplementary Material

Table S1 shows clinical data of NAFLD/NASH patients.

Table S2 shows molecular characteristics of chemically modified βCD-PRXs.

Table S3 shows serological parameters of WT mice treated with chemically modified βCD-PRXs.

Table S4 shows serum cytokine levels in MC4R-KO mice treated with βCD-PRX for 6 wk.

Table S5 shows effect of 6-wk βCD-PRX treatment on serological parameters of WT mice fed an HC diet for 20 wk.

Table S6 shows primers used in this study.

SourceData FS4 is the source file for Fig. S4.

## Data Availability

The RNA-seq data are available on the website of the Gene Expression Omnibus at the National Center for Biotechnology Information (GEO accession no. GSE235024 and GSE235222).
