# Supplementary material for: Lysosomal cholesterol overload in macrophages promotes liver fibrosis in a mouse model of NASH
Source: J Exp Med. 2023 Sep 19;220(11):e20220681. doi: 10.1084/jem.20220681 (PMC10506914; doi:10.1084/jem.20220681)
Supplement: Table S1 — shows clinical data of NAFLD/NASH patients. [file JEM_20220681_TableS1.docx]

**Table S1. Clinical data of NAFLD/NASH patients**

Spearman’s rank

correlation coefficient

*Clinical information* mean with CLS number

Sex (M/F) 49/49 -0.220

Age 55.5 ± 1.56 -0.311

Body mass index 28.0 ± 0.43 0.284

*Blood parameters*

Platelet (x10^4/μl) 21.1 ± 0.59 0.261

Aspartate aminotransferase (IU/liter) 46.3 ± 2.25* 0.356

Alanine aminotransferase (IU/liter) 69.0 ± 4.51* 0.505

γ-glutamyl transpeptidase (IU/liter) 72.7 ± 6.90* 0.248

Total bilirubin (mg/dl) 0.84 ± 0.07 -0.032

Albumin (g/dl) 4.41 ± 0.04 0.286

Total cholesterol (mg/dl) 198.4 ± 4.7 0.297

Triglyceride (mg/dl) 158.7 ± 11.3* 0.259

Free fatty acid (mEq/liter) 0.97 ± 0.78* 0.027

High density lipoprotein (mg/dl) 51.8 ± 1.52 -0.211

Low density lipoprotein (mg/dl) 122.4 ± 3.62 0.387

Free cholesterol (mg/dl) 50.7 ± 1.28 0.295

Apolipoprotein A-I (mg/dl) 130.4 ± 2.34 -0.242

Apolipoprotein B (mg/dl) 100.7 ± 2.56 0.439

Apolipoprotein E (mg/dl) 5.32 ± 0.31 0.172

Remnant cholesterol (mg/dl) 15.4 ± 4.28 0.449

Fasting blood glucose (mg/dl) 118.4 ± 2.3* 0.141

Fasting insulin (μU/ml) 15.5 ± 0.67* 0.386

HbA1c (%) 6.39 ± 0.10* 0.147

High sensitive C-responsive protein (mg/dl) 0.23 ± 0.04 0.086

Hyaluronic acid (ng/ml) 55.2 ± 6.31* -0.127

Ferritin (ng/ml) 235.4 ± 20.4 0.242

Fe (μg/dl) 127.0 ± 4.1 0.145

FIB-4 index 1.75 ± 0.17* -0.222

*Histological scores*

Steatosis 1.61 ± 0.08 0.513

Inflammation 1.17 ± 0.05 0.216

Ballooning 0.30 ± 0.05 0.306

NAFLD activity score 3.05 ± 0.14 0.502

Fibrosis 1.62 ± 0.10 -0.059

*Imaging measurements*

Liver stiffness measurement (kPa) 15.65 ± 3.64* -0.070

Controlled attenuation parameter (dB/m) 283.5 ± 5.71* 0.518

* Above the normal range.
