# Supplementary material for: Lysosomal cholesterol overload in macrophages promotes liver fibrosis in a mouse model of NASH
Source: J Exp Med. 2023 Sep 19;220(11):e20220681. doi: 10.1084/jem.20220681 (PMC10506914; doi:10.1084/jem.20220681)
Supplement: Table S2 — shows molecular characteristics of chemically modified βCD-PRXs. [file JEM_20220681_TableS2.docx]

**Table S2. Molecular characteristics of chemically modified βCD-PRXs**

Chemically modified number of number of modification Average molecular

βCD-PRX threading βCD groups (/βCD-PRX) weight

Me-βCD-PRX 15.2 8.50 (129) 25,400

Ac-βCD-PRX 15.2 3.65 (55.5) 25,900

HEE-βCD-PRX 15.2 5.78 (87.9) 35,100

MEEE-βCD-PRX 15.2 5.75 (84.7) 39,600

CM-βCD-PRX 12.3 3.12 (38.4) 28,200

DMAE-βCD-PRX 15.2 4.67 (71.0) 34,000

SPAE-βCD-PRX 15.2 4.67 (71.0) 42,700

Me, methyl; Ac, acetyl; HEE, 2-(2-hydroxyethoxy)ethyl carbamate; MEEE, 2-(2-(2-methoxyethoxy)ethoxy)ethyl carbamate; CM, carboxymethyl carbamate; DMAE, 2-(*N*,*N*-dimethylamino)ethyl carbamate; SPAE, 2-(*N*-3-Sulfopropyl-*N*,*N*-dimethylammonium)ethyl carbamate.
