# Supplementary material for: Lysosomal cholesterol overload in macrophages promotes liver fibrosis in a mouse model of NASH
Source: J Exp Med. 2023 Sep 19;220(11):e20220681. doi: 10.1084/jem.20220681 (PMC10506914; doi:10.1084/jem.20220681)
Supplement: Table S3 — shows serological parameters of WT mice treated with chemically modified βCD-PRXs. [file JEM_20220681_TableS3.docx]

**Table S3. Serological parameters of wild-type mice treated with chemically modified βCD-PRXs.**

Chemical

modification Veh Me Ac HEE MEEE CM DMAE SPAE

AST (U/L) 306 ± 84.3 223 ± 39.3 250 ± 47.3 166 ± 13.2 122 ± 14.0 226 ± 35.2 541 ± 35.2* 168 ± 58.2

ALT (U/L) 31 ± 2.6 25 ± 2.5 28 ± 2.9 25 ± 2.9 21 ± 1.1 68 ± 40.7 123 ± 14.6** 23 ± 1.1

ALB (g/dl) 2.24 ± 0.08 2.28 ± 0.09 2.38 ± 0.11 2.26 ± 0.10 2.16 ± 0.02 2.34 ± 0.04 2.50 ± 0.06 2.20 ± 0.06

T-Bil (mg/dl) 0.46 ± 0.02 0.42 ± 0.02 0.32 ± 0.02 0.34 ± 0.02 0.44 ± 0.02 0.42 ± 0.02 0.54 ± 0.09 0.40 ± 0.00

ALP (U/L) 225 ± 6.9 210 ± 8.0 206 ± 6.5 222 ± 14.3 197 ± 5.9 217 ± 5.1 307 ± 16.2** 202 ± 10.4

AST, aspartate aminotransferase; ALT, alanine aminotransferase; ALB, albumin; T-Bil, total bilirubin; ALP, alkaline phosphatase. *n* = 5. ** *P* < 0.01, *P* < 0.05 vs. veh. Data are representative of two independent experiments. Data are expressed as the mean ± SEM.
