# Supplementary material for: Lysosomal cholesterol overload in macrophages promotes liver fibrosis in a mouse model of NASH
Source: J Exp Med. 2023 Sep 19;220(11):e20220681. doi: 10.1084/jem.20220681 (PMC10506914; doi:10.1084/jem.20220681)
Supplement: Table S4 — shows serum cytokine levels in MC4R-KO mice treated with βCD-PRX for 6 wk. [file JEM_20220681_TableS4.docx]

**Table S4. Serum cytokine levels in MC4R-KO mice treated with βCD-PRX for 6 weeks**

WT/SD MC/WD

Cont Cont PRX

TNFα (pg/ml) 0.44 ± 0.09 6.18 ± 0.09** 7.33 ± 1.19**

IL-1β (pg/ml) 6.24 ± 0.26 7.10 ± 0.44** 7.05 ± 0.11**

MCP-1 (pg/ml) 48.2 ± 10.9 285.5 ± 30.0** 200.4 ± 23.3**#

Osteopontin (ng/ml) 2.023 ± 0.07 4.78 ± 0.26** 3.93 ± 0.35**#

PDGF-BB (ng/ml) 1.51 ± 0.08 2.01 ± 0.16** 2.13 ± 0.07**

WT, wild-type mice; SD, standard diet; MC, melanocortin 4 receptor–deficient mice; WD, Western diet; Cont, control; PRX, β-cyclodextrin polyrotaxane; TNFα, tumor necrosis factor-α; MCP-1, monocyte chemoattractant protein-1; IL-1β, interleukin-1β; PDGF-BB, platelet-derived growth factor-BB. *n* = 4-7. ** *P* < 0.01 vs WT/SD-Cont, # *P* < 0.05 vs. MC/WD-Cont. Data are representative of two independent experiments. Data are expressed as the mean ± SEM.
