# Supplementary material for: Lysosomal cholesterol overload in macrophages promotes liver fibrosis in a mouse model of NASH
Source: J Exp Med. 2023 Sep 19;220(11):e20220681. doi: 10.1084/jem.20220681 (PMC10506914; doi:10.1084/jem.20220681)
Supplement: Table S5 — shows effect of 6-wk βCD-PRX treatment on serological parameters of WT mice fed an HC diet for 20 wk. [file JEM_20220681_TableS5.docx]

**Table S5. Effect of 6-week βCD-PRX treatment on serological parameters of wild-type mice fed a high-cholesterol diet for 20 weeks.**

SD HC

Cont Cont PRX

BG (mg/dl) 118.5 ± 5.2 184.7 ± 6.3 187.5 ± 9.3

TG (mg/dl) 88.5 ± 5.3 72.0 ± 6.6 64.7 ± 3.3

TC (mg/dl) 73.5 ± 2.9 306.7 ± 17.4** 273.7 ± 10.3

AST (U/liter) 200.2 ± 33.1 413.4 ± 35.5** 398.5 ± 40.5**

ALT (U/liter) 68.3 ± 23.8 456.9 ± 68.6** 378.8 ± 81.1**

Hepatic TG (μg/mg tissue) 3.8 ± 0.3 173.5 ± 9.3** 176.8 ± 11.9**

Hepatic TC (μg/mg tissue) 2.6 ± 0.1 11.5 ± 0.8** 13.6 ± 0.9**

HC, high-cholesterol diet; BG, blood glucose; TG, tryglyceride; TC, total cholesterol; AST, aspartate aminotransferase; ALT, alanine aminotransferase. SD-Cont, *n* = 6; HC-Cont, *n* = 7; HC-PRX, *n* = 6. ** *P* < 0.01 vs. SD-Control. Data are expressed as the mean ± SEM.
