# Supplementary material for: Lysosomal cholesterol overload in macrophages promotes liver fibrosis in a mouse model of NASH
Source: J Exp Med. 2023 Sep 19;220(11):e20220681. doi: 10.1084/jem.20220681 (PMC10506914; doi:10.1084/jem.20220681)
Supplement: Table S6 — shows primers used in this study. [file JEM_20220681_TableS6.docx]

**Table S6. Primers used in this study.**

| Gene name | Primer | Sequence | Product size (bp) |
| --- | --- | --- | --- |
| *36B4* | forward | 5'-GGCCCTGCACTCTCGCTTTC-3' | 124 |
|  | reverse | 5'-TGCCAGGACGCGCTTGT-3' |  |
| *Abca1* | forward | 5'-ATTGCCAGACGGAGCCG-3' | 103 |
|  | reverse | 5'-TGCCAAAGGGTGGCACA-3' |  |
| *Abcg1* | forward | 5'-TTCGCTGCTCTGGGTACCA-3' | 108 |
|  | reverse | 5'-TGTCACGGGACCCACAAAT-3' |  |
| *Acc1* | forward | 5'-TGAGATTGGCATGGTAGCCTG-3' | 62 |
|  | reverse | 5'-CTCGGCCATCTGGATATTCAG-3' |  |
| *Acox* | forward | 5'-GCCTTTGTTGTCCCTATCCGT-3' | 70 |
|  | reverse | 5'-CGATATCCCCAACAGTGATGC-3' |  |
| *Atp6v0d2* | forward | 5'-CAAAGCCAGCCTCCTAACTCA-3' | 76 |
|  | reverse | 5'-ATGAATTTTCAGGTCTTCCAAGGT-3' |  |
| *Ccl2* | forward | 5'-CCACTCACCTGCTGCTACTCAT-3' | 76 |
|  | reverse | 5'-TGGTGATCCTCTTGTAGCTCTCC-3' |  |
| *Ccl3* | forward | 5'-CTGTTCTTCTCTGTACCATGACACTCT3' | 54 |
|  | reverse | 5'-CATATGGCGCTGAGAAGACTTG-3' |  |
| *Cd36* | forward | 5'-CCAAATGAAGATGAGCATAGGACA-3' | 51 |
|  | reverse | 5'-TCCAGTTATGGGTTCCACATCTAAG-3' |  |
| *Chop* | forward | 5'-CCACCACACCTGAAAGCAGAA -3' | 67 |
|  | reverse | 5'-AGGTGAAAGGCAGGGACTCA-3' |  |
| *Col1a1* | forward | 5'-CCTCAGGGTATTGCTGGACAAC-3' | 126 |
|  | reverse | 5'-ACCACTTGATCCAGAAGGACCTT-3' |  |
| *Col4a1* | forward | 5'-GGCGGTACACAGTCAGACCAT-3' | 89 |
|  | reverse | 5'-TGGTGTGCATCACGAAGGAA-3' |  |
| *Cpt1a* | forward | 5'-CCTGCATTCCTTCCCATTTG-3' | 116 |
|  | reverse | 5'-TGCCCATGTCCTTGTAATGTG-3' |  |
| *Ctgf* | forward | 5'-GCCCTAGCTGCCTACCGACT-3' | 51 |
|  | reverse | 5'-CATAGTTGGGTCTGGGCCAA-3' |  |
| *Ctsd* | forward | 5'-ACATCCACTACGGCTCAGGAA-3' | 73 |
|  | reverse | 5'-TCAGACTTACATGGAACCGATACAG-3' |  |
| *Egr1* | forward | 5'-GACGAGTTATCCCAGCCAAA-3' | 202 |
|  | reverse | 5'-GGCAGAGGAAGACGATGAAG-3' |  |
| *Fasn* | forward | 5'-CCTGGATAGCATTCCGAACCT-3' | 122 |
|  | reverse | 5'-AGCACATCTCGAAGGCTACACA-3' |  |
| *G6pase* | forward | 5'-CACCTGTGAGACCGGACCA-3' | 73 |
|  | reverse | 5'-GACCATAACATAGTATACACCTGCTGC-3' |  |
| *Gadd34* | forward | 5'-CCCTCCAACTCTCCTTCTTCAG-3' | 139 |
|  | reverse | 5'-CAGCCTCAGCATTCCGACAA-3' |  |
| *Gpnmb* | forward | 5'-GTACAAAAAACACAAGGCGTACAA-3' | 90 |
|  | reverse | 5'-GCGTGACTGAGGAGAACACTCA-3' |  |
| *Grp78* | forward | 5'-GTTCTTGCCATTCAAGGTGG-3' | 181 |
|  | reverse | 5'-TGGTACAGTAACAACTGCATG-3' |  |
| *Hmgcr* | forward | 5'-CCAAACCCCGTAACCCAAA-3' | 71 |
|  | reverse | 5'-CGACTATGAGCGTGAACAAGGA-3' |  |
| *Hmgcs* | forward | 5'-TCTTGGGATGGACGATATGCT-3' | 66 |
|  | reverse | 5'-GGCATTTCCTGTGGCATATATAGC-3' |  |
| *Il1β* | forward | 5'-CTGGTGTGTGACGTTCCCATTA-3' | 76 |
|  | reverse | 5'-CCGACAGCACGAGGCTTT-3' |  |
| *Il6* | forward | 5'-ACAACCACGGCCTTCCCTACTT-3' | 129 |
|  | reverse | 5'-CACGATTTCCCAGAGAACATGTG-3' |  |
| *Il10* | forward | 5'-AATAAGCTCCAAGACCAAGGTGTC-3' | 51 |
|  | reverse | 5'-GATGTCAAATTCATTCATGGCCT-3' |  |
| *Itgax* | forward | 5'-GCCATTGAGGGCACAGAGA-3' | 66 |
|  | reverse | 5'-GAAGCCCTCCTGGGACATCT-3' |  |
| *Kcnn4* | forward | 5'-GCCAGGTACGGCTGAAACA-3' | 79 |
|  | reverse | 5'-AGGATCATGTGCATCTTGGAGAT-3' |  |
| *Ldlr* | forward | 5'-GCGGCTTCCGGTTGGT-3' | 78 |
|  | reverse | 5'-AGAGCTGGCTGCAGGTGTCT-3' |  |
| *Msr1* | forward | 5'-GAAATTTGACGCACGTTCAATG-3' | 51 |
|  | reverse | 5'-TTTTTAGTGCTGTGAGGAAGGGAT-3' |  |
| *Mttp* | forward | 5'-ACAGGTCCTCGAGCGTGTCT-3' | 51 |
|  | reverse | 5'-CAGTGCTCCGCCAGAGAAG-3' |  |
| *Nr1h3* | forward | 5'-CTCAATGCCTGATGTTTCTCCT-3' | 150 |
|  | reverse | 5'-TCCAACCCTATCCCTAAAGCAA-3' |  |
| *Nr1h2* | forward | 5'-CCCCACAAGTTCTCTGGACACT-3' | 70 |
|  | reverse | 5'-TGACGTGGCGGAGGTACTG-3' |  |
| *Pdgfb* | forward | 5'-CCATCCGCTCCTTTGATGAT-3' | 67 |
|  | reverse | 5'-TCAGCCCCATCTTCATCTACG-3' |  |
| *Pfk* | forward | 5'-GGCCAATCCTCAAAATCCTA-3' | 91 |
|  | reverse | 5'-CCAGACCGTTTCCTTGAAAT-3' |  |
| *Ppara* | forward | 5'-AGGAAGCCGTTCTGTGACAT-3' | 171 |
|  | reverse | 5'-AATCCCCTCCTGCAACTTCT-3' |  |
| *Spp1* | forward | 5'-CCATCTCAGAAGCAGAATCTCC-3' | 132 |
|  | reverse | 5'-ATCGTCATCATCGTCGTCC-3' |  |
| *Srebf1* | forward | 5'-AGCTGTCGGGGTAGCGTCTG-3' | 187 |
|  | reverse | 5'-GAGAGTTGGCACCTGGGCTG-3' |  |
| *Srebf2* | forward | 5'- CAGCTGCTGGAGCATAGCCT-3' | 121 |
|  | reverse | 5'- GAAGATGGCGGCAGGCCAG-3' |  |
| *Tfe3* | forward | 5'-CCACACACTGAGTCGTCCAC-3' | 72 |
|  | reverse | 5'-TTCTCGAGGTGGGTCTGAAC-3' |  |
| *Tfeb* | forward | 5'-CCTGCCGACCTGACTCAGA-3' | 97 |
|  | reverse | 5'-TCTCAATTAGGTTGTGATTGTCTTTCTT-3' |  |
| *Tgfb1* | forward | 5'-CCTGAGTGGCTGTCTTTTGACG-3' | 91 |
|  | reverse | 5'-AGTGAGCGCTGAATCGAAAGC-3' |  |
| *Timp1* | forward | 5'-CATCACGGGCCGCCTA-3' | 51 |
|  | reverse | 5'-AAGCTGCAGGCACTGATGTG-3' |  |
| *Tnf* | forward | 5'-ACCCTCACACTCAGATCATCTTC-3' | 71 |
|  | reverse | 5'-TGGTGGTTTGCTACGACGT-3' |  |
| *Vegfa* | forward | 5'-TGAACACCAGCACAGGTTACCT-3' | 130 |
|  | reverse | 5'-GCCTTGTGAGAGAGGCACTGTA-3' |  |
| *Xbp1s* | forward | 5'-GAGTCCGCAGCAGGTG-3' | 65 |
|  | reverse | 5'-GTGTCAGAGTCCATGGGA-3' |  |
