# Supplementary figures and images for: Lysosomal cholesterol overload in macrophages promotes liver fibrosis in a mouse model of NASH
Source: J Exp Med. 2023 Sep 19;220(11):e20220681. doi: 10.1084/jem.20220681 (PMC10506914; doi:10.1084/jem.20220681)

LC-3

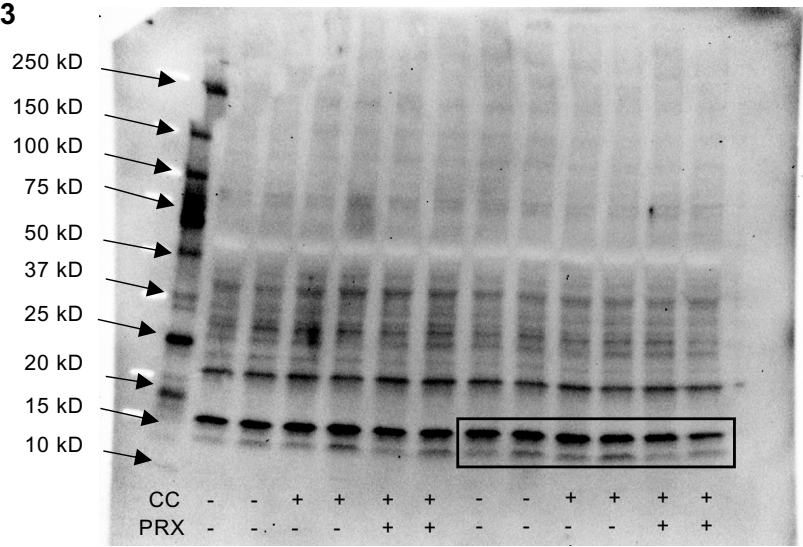

p62

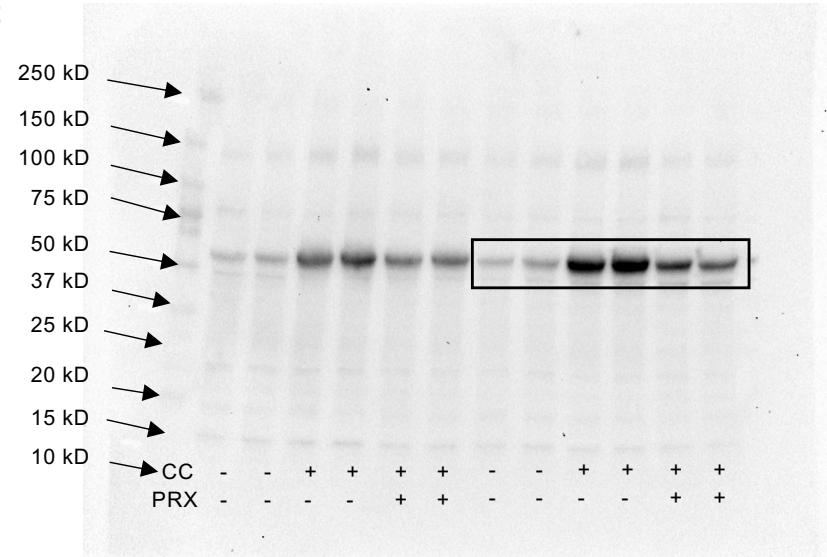

$\alpha$ -tubulin

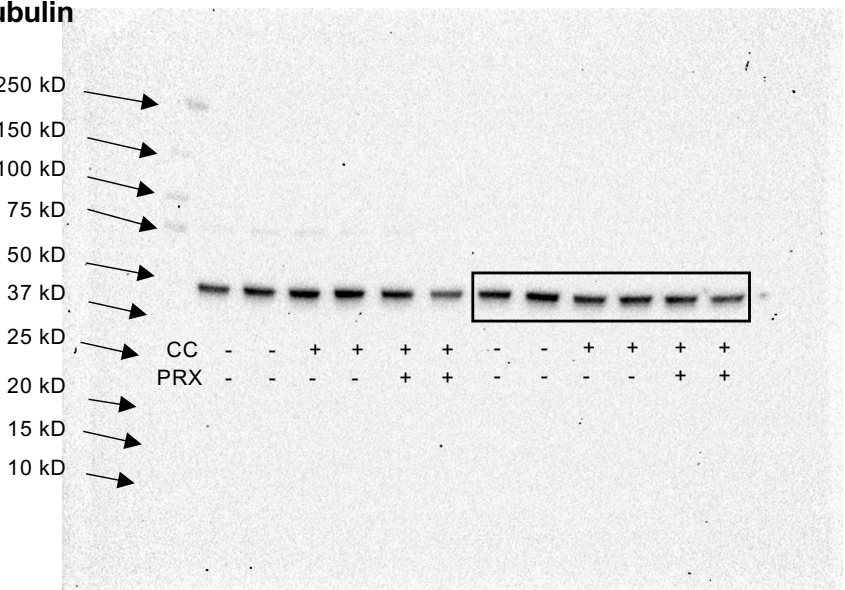

Supplement: SourceData FS4 — is the source file for Fig. S4. [file JEM_20220681_SourceDataFS4.pdf]
